# From *Sophora japonica* to Smart Nanomedicine: Molecular Docking Simulations and Multifaceted Applications of CaO Nanoparticles

**DOI:** 10.1021/acsomega.5c03710

**Published:** 2025-10-10

**Authors:** Ecem Baykal Alpaslan, Azade Attar, Emre Aktas, Melda Altikatoglu Yapaoz

**Affiliations:** † Yildiz Technical University, Faculty of Science and Letters, Department of Chemistry, Davutpasa Campus, Istanbul 34220, Turkey; ‡ Istanbul Gelisim University, Vocational School Of Health Services, Medical Laboratory Techniques Program, Avcilar Campus, Istanbul 34310, Turkey; § Yildiz Technical University, Faculty of Chemical & Metallurgical Engineering, Department of Bioengineering, Davutpasa Campus, Istanbul 34220, Turkey; ∥ Yildiz Technical University, Faculty of Science and Letters, Department of Molecular Biology and Genetics, Davutpasa Campus, Istanbul 34220, Turkey

## Abstract

The growing demand for multifunctional nanomaterials in biomedical and environmental applications has driven the need for sustainable synthesis methods and comprehensive performance evaluations. In this study, calcium oxide nanoparticles (CaONPs) were synthesized using *Sophora japonica* extract via a green route, comprehensively characterized, and evaluated for biomedical and environmental applications. UV–vis spectroscopy confirmed the formation of CaONPs with a characteristic absorption peak at 321 nm. SEM showed spherical morphology with an average size of 30–70 nm, and FT-IR analysis confirmed the removal of organic residues postcalcination. X-ray diffraction analysis revealed sharp peaks corresponding to crystalline CaO with an average crystallite size of 53.45 nm. Molecular docking simulations were performed to evaluate the binding potential of synthesized CaONPs against selected bacterial outer membrane proteins (7NG9, 1BY3, 1FEB, 2HDF, and 4C4V) and the FDPS enzyme. The results revealed that CaO exhibited strong and stable binding interactions, comparable to or exceeding those of reference drugs, suggesting its promise as a dual-function bioactive agent. The calcinated CaONPs exhibited notable antibacterial and antifungal activity, with inhibition zones up to 18 mm, which enhanced up to 27 mm in combination with antibiotics/antifungals. In drug delivery studies, Zoledronic acid-loaded CaONPs showed pH-responsive behavior, releasing 92% of the drug at 250 h at pH 5.0, suggesting targeted delivery potential in acidic tumor environments. CaONPs showed no toxicity to Saos-2 osteosarcoma cells with 82% cell viability at 500 μg/mL and 78% cell viability at 1000 μg/mL. Furthermore, CaONPs achieved 93% removal efficiency of Congo red at 50 °C and pH 5.0 in 24 h, highlighting their potential in wastewater treatment. Synthesized CaONPs exhibited antimicrobial, drug delivery, and dye degradation properties while maintaining biocompatibility. Their pH-dependent drug release performance and strong synergistic antimicrobial effects highlight their applicability in antibiotic resistance, cancer therapy, and wastewater treatment.

## Graphical abstract

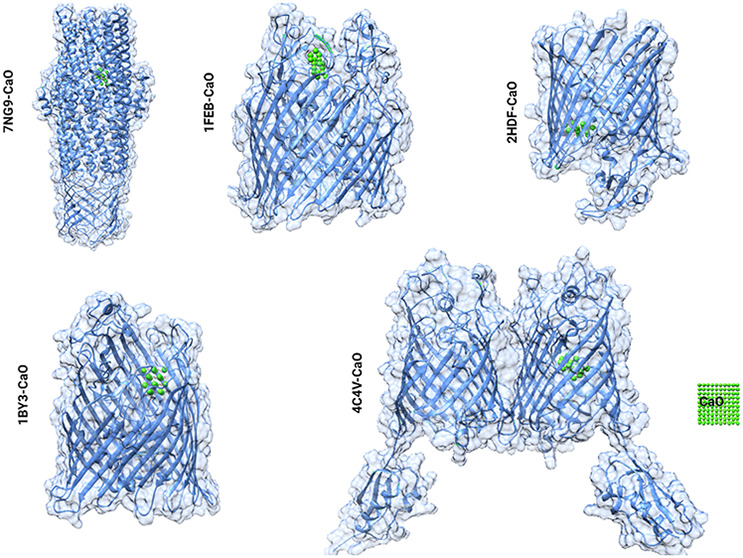

## 1 Introduction

Since the emergence of the concept of green chemistry, the construction of natural products from plant sources in an effective, environmentally friendly, and sustainable manner has become the main goal in the application of natural resources. The use of plant extracts in the synthesis of nanoparticles has proven to be cost-effective and has opened up a wide field for the synthesis of nontoxic nanoparticles. One of the current examples of these applications, calcium oxide (CaO) nanoparticles, has applications in areas such as catalysis, adsorption, water treatment, and also antibacterial agents. Since CaO is considered a safe material for humans and animals, the use of nanoparticles in such areas is of particular interest. CaO is an important inorganic compound in nanoparticle applications that can be employed as a catalyst,^1^ pellets for CO_2_ capture and kinetic tests,^2^ waste remediation vector or an ingredient in refractory and paint industries,^3^ antimicrobial agent, a drug delivery vehicle, and also in other biomedical applications.^4^ CaO has been considered as one of the most favorable examples of metal nanoparticles for carbon capture due to its good kinetic properties, high capture capacity, and low operating cost, even under low CO_2_ partial pressures.^5^ Nanoscale CaO offers a much greater reactive surface area, increasing the number of active sites for CO_2_ adsorption and reaction. This improves the reaction kinetics, enabling faster CO_2_ uptake. Various methods, such as sol–gel, thermal decomposition, hydrothermal technique, combustion method, coprecipitation technique, biogenic method, precipitation method, two-step thermal decomposition technique, single-step multicomponent synthesis, and microwave synthesis, can be used to prepare CaO nanoparticles. By changing these methods, many physical and chemical properties of CaO nanoparticles, including morphology, specific surface area, and entrapment efficiency, can be changed.^6^

Green synthesis, which has become increasingly important, especially in nanoparticle synthesis, has been used since the early 2000s and is an important synthesis method that increases sustainability by reducing environmental impact. The main purpose of these synthesis methods is to prefer chemicals with lower toxicity than normal or no toxic properties and, at the same time, to reduce energy consumption as much as possible. In addition, green synthesis methods also aim to reduce the amount of waste that may be generated or to obtain useful products by evaluating the waste. Therefore, these approaches in green synthesis processes allow chemical reactions in industrial processes to be carried out both more efficiently and cleaner.^7^ Therefore, in recent years, the synthesis and characterization of nanoparticles from different plant species have taken their place in the literature as an interesting field. *Sophora japonica* used in this study is one of them. *Sophora japonica* L. is a perennial, deciduous, woody plant belonging to the genus *Sophora* in the family *Leguminosae*. Considering its first discovery, it was later globally cultivated especially in Asia and Europe after its occurrence in China.^8^ *S. japonica* is a significant economic plant recognized for its edible and medicinal attributes. Its flowers and flower buds are utilized in a variety of food applications, including dishes, dumplings, cakes, and beverages. Additionally, extracts from *S. japonica* serve multiple purposes in the food industry, such as acting as a preservative in sausages, enhancing color and flavor in rice wine, functioning as a synergist in low-fat yogurt, and providing antioxidant properties in edible films.^9,10^ Furthermore, polysaccharides derived from its seeds have applications as food hydrocolloids. Beyond its culinary uses, different parts of *S. japonica*, including flowers, fruits, bark, branches, and leaves, have been integral to traditional medicine for over two millennia. They have been employed for their therapeutic effects, such as reducing heat, detoxifying the body, controlling bleeding, and lowering blood pressure.^11^ Contemporary pharmacological studies highlight the plant’s broad spectrum of biological activities, including antibacterial, antioxidant, anti-inflammatory, anticancer, and cardioprotective effects.^12^ The bioactive compounds in *S. japonica*, particularly its reducing agents, contribute to various health benefits, such as anti-inflammatory, antibacterial, antiviral, antioxidant, free radical-scavenging, and antihyperglycemic properties.^13^ The plant’s health-promoting effects are closely linked to its diverse phytochemical composition, which includes flavonoids, triterpenoids, polysaccharides, volatile oils, fatty acids, amino acids, and trace elements. Notably, flavonoids are prevalent throughout all parts of the plant.^14^ While previous reviews have primarily explored the traditional applications, phytochemistry, and pharmacological effects of *S. japonica*, recent studies have also investigated its role in the synthesis of nanoparticles, including sulfur, zinc oxide, and copper oxide.^15–17^

In addition to all of the mentioned properties, CaO nanoparticles (CaONPs) may be useful in drug delivery and release due to their biocompatibility, so it is necessary to investigate the cytotoxicity of synthesized CaONPs. In the study, CaONPs were synthesized using the fruit extract of the *S. japonica*, and the nanoparticles were characterized using UV–vis, FT-IR, XRD, SEM, and Zeta potential analysis. The antibacterial and antifungal activities of the synthesized nanoparticles were investigated against *Escherichia coli*, *Staphylococcus aureus*, *Aspergillus niger*, and *Candida albicans*, and dye removal was studied using Congo red. The cytotoxicity of CaONPs was tested on the Saos-2 bone cell line using MTT analysis. *In vitro* drug release studies were performed by using Zoledronic acid. CaONPs were studied to reveal their binding affinities with selected bacterial outer membrane proteins—Colistin, Cefiderocol, Darobactin, Gallium maltolate, and Carbonyl Cyanide m-Chlorophenyl Hydrazone—using molecular docking analysis, for the first time reported in the literature. Molecular docking simulations were also obtained with the protein target, Farnesyl diphosphate synthase (FDPS), which is a crucial enzyme in the mevalonate pathway that regulates isoprenoid biosynthesis, necessary for osteoclast function and bone resorption.^18^ Due to its central role in osteoclast activity, FDPS has been widely recognized as the molecular target of nitrogen-containing bisphosphonates, including Zoledronic acid.^19^ In this study, green synthesis of CaONPs from *S. japonica*, their potential as an effective alternative against antibiotic resistance when used in combination with antibacterial and antifungal drugs, and *in vitro* pH-dependent controlled release of Zoledronic acid by CaONPs and molecular docking simulations were demonstrated for the first time in the literature. Overall, the study provides valuable insights into the synthesis and characterization of CaONPs, demonstrating their multifunctional potential in antimicrobial applications, dye degradation, drug delivery, and biomedical uses, paving the way for their further optimization and large-scale biomedical and environmental applications.

## 2 Materials and Methods

### 2.1 Synthesis of CaONPs

The fruits of *S. japonica* collected from the Yildiz Technical University, Davutpasa Campus, were washed with distilled water and left to dry in a 60 °C oven. Dried fruits were blended and ground into powder. 10 g of plant powder was incubated in 100 mL of pure water at 80 °C for 45 min at 125 rpm. The solution was used in the preparation of mixtures in which the ratios between 0.1 M CaCl_2_ and plant extract were 1:5, 1:2, 1:1, 2:1, and 5:1 (v/v). These solutions at different ratios were stirred in a 60 °C water bath at 125 rpm for 2 h. In order to investigate the effect of pH on nanoparticle formation, 0.1 M NaOH was added in amounts varying between 0.5 and 2.5 mL, and the mixture was centrifuged. The precipitates obtained after the washing process were left to dry in a 50 °C oven. The nanoparticles obtained at the end of this step were coded as **CaONP I**. In addition, a calcination process was applied to the samples. CaCl_2_ and *S. japonica* nanoparticles prepared at 5:1 (v/v), the rate at which the highest amount of nanoparticles was obtained, were subjected to a calcination process in an oven at 500 °C for 3 h. Nanoparticles obtained at the end of this process are coded as **CaONP II**. Scheme 1 outlines the steps involved in the synthesis of nanoparticles. In this study, the two types of CaONPs before and after calcination were analyzed and compared.

**Scheme 1:**
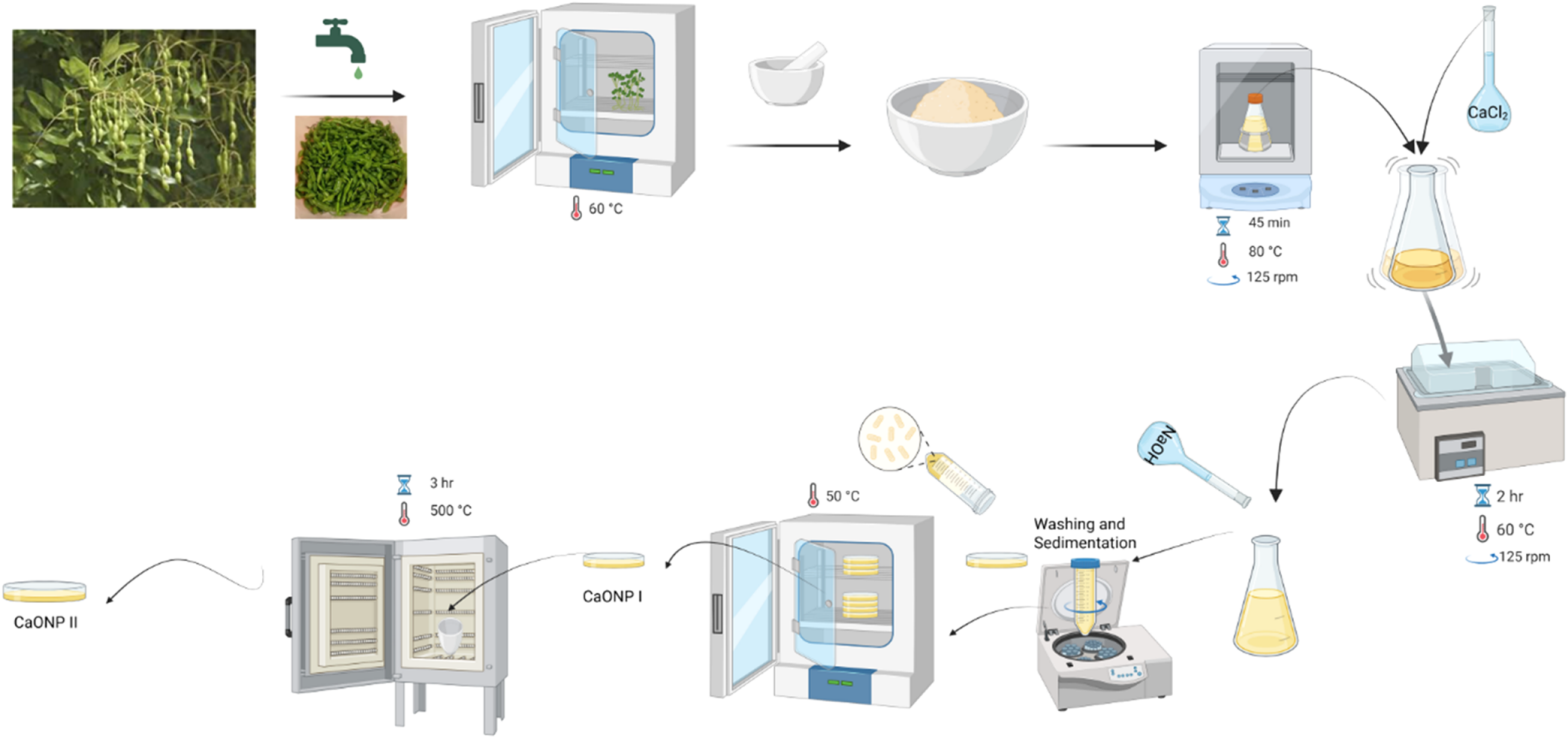
An Overview of the CaO Nanoparticle Synthesis Process^a^

a Created in BioRender; BAYKAL ALPASLAN, E. (2025) https://BioRender.com/c7n4by0.

### 2.2 Characterization

CaONPs and *S. japonica* extract were characterized with various analyses. UV–vis spectra were measured between 200 and 800 nm wavelengths before and after calcination using a Shimadzu brand UV2600 model spectrophotometer. FT-IR analyses were carried out on a Thermo brand Nicolet IS 10 model spectrophotometer in the 4000–650 nm wavelength range. UV–vis and IR spectra were obtained both before and after calcination. Characterization with SEM was carried out on a Zeiss brand EVO LS 10 model device. The Malvern PANalytical brand X’Pert PRO model XRD device was used in XRD analysis. The Malvern Panalytical brand Zetasizer Nano ZS was used in the zeta potential measurement.

### 2.3 Construction of CaONP Surface by Molecular Docking Analysis

The crystalline structure of calcium oxide (CaO) was constructed using the ASE (atomic simulation environment) Python package.^20^ A bulk rock-salt-type CaO structure was initially generated with a lattice parameter of 4.81 Å, reflecting the experimental crystallographic spacing of CaO. To create a representative nanoparticle surface suitable for molecular docking studies, the bulk unit cell was replicated 4 × 4 × 1 times along the *x*, *y*, and *z* axes, respectively, resulting in a slab-like structure. The resulting model preserves the crystallographic integrity while providing sufficient surface exposure for reliable docking simulations. The finalized CaO surface structure was saved in pdb format and used as the receptor model for comparative binding analyses with clinically validated ligands.

### 2.4 Preparation and Docking of Ligands with Target Proteins: Nanoparticle Versus Drug Comparisons

#### 2.4.1 Preparation of Zoledronic Acid and Its Target Protein Farnesyl Diphosphate Synthase for Molecular Docking

The chemical structures of Zoledronic acid (PubChem CID: 68740) were retrieved from the PubChem database and imported into UCSF Chimera 1.18 for preparation.^21^ For each ligand, hydrogen atoms were added by using the “AddH” tool, treating each molecule individually and enabling hydrogen bond optimization. Gasteiger charges were assigned, and the net charges were adjusted according to molecular configurations. Energy minimization was conducted using steepest descent (100 steps) and conjugate gradient (10 steps) methods with a step size of 0.02 Å for both, ensuring energetically stable conformations. Ligand files were saved in both .mol2 and .pdbqt formats for subsequent docking. The protein target, Farnesyl diphosphate synthase (FDPS, PDB ID: 1ZW5), was downloaded from the RCSB Protein Data Bank. FDPS has been widely recognized as the molecular target of nitrogen-containing bisphosphonates, including Zoledronic acid.^19^ Therefore, it was chosen as the docking receptor to assess the binding potential of the ligands. All nonprotein components, such as water molecules and heteroatoms, were removed, and the protein structure underwent energy minimization in Chimera to relieve any steric clashes or conformational strain. The minimized protein structure was then prepared for docking in AutoDock Vina by assigning appropriate charges and saving the file in .pdbqt format.^22^

#### 2.4.2 Ligands and Outer Membrane Proteins Preparation for Comparative Molecular Docking

To evaluate the binding affinities of clinically relevant antibiotics and small molecules toward bacterial outer membrane proteins (OMPs), a panel of validated ligand–protein pairs was selected based on evidence from the literature.^23–27^ These interactions were further analyzed to compare their binding properties to those of synthesized CaONPs in silicon. The chemical structures of known ligands, including Colistin (CID: 5311054), Cefiderocol (CID: 77843966), Darobactin (CID: 154586077), Gallium maltolate (CID: 9846339), and Carbonyl Cyanide m-Chlorophenyl Hydrazone (CCCP: CID: 2603), were retrieved from the PubChem database. Each ligand was imported into UCSF Chimera 1.18 for the structure preparation. Hydrogen atoms were added using the “AddH” function, and Gasteiger partial charges were assigned. To ensure accurate ligand geometry, energy minimization was conducted using the steepest descent algorithm (100 steps), followed by the conjugate gradient method (10 steps), both with a step size of 0.02 Å. The minimized structures were saved in both .mol2 and .pdbqt formats to ensure compatibility with AutoDock Vina.

Corresponding bacterial outer membrane protein targets were retrieved from the RCSB Protein Data Bank (PDB) using their validated PDB IDs: LptD (4RHB), FepA (1FEP), CirA (2HDF), FhuA (1BY3), BamA (4C4V), and TolC (7NG9). These proteins were selected based on their functional roles in drug transport, membrane stability, and antibiotic resistance mechanisms. All nonprotein components such as water molecules, ions, and heteroatoms were removed upon import into UCSF Chimera 1.18. Each protein structure was subjected to energy minimization to relieve steric clashes and improve structural geometry using Chimera’s built-in optimization tools. Following minimization, polar hydrogens and appropriate charges were added, and each structure was saved in .pdbqt format for molecular docking. Molecular docking studies were then conducted using AutoDock Vina to quantitatively compare the binding affinities of the ligands to their respective protein receptors against those of the synthesized CaONPs. This comparative approach aimed to determine whether the nanoparticles exhibit competitive or superior binding behavior relative to known antibacterial agents at their validated membrane-associated targets.

To validate the docking protocol, a redocking process was carried out using the cocrystallized ligand of the reference protein (FDPS, PDB ID: 1ZW5). The native ligand was removed and redocked into the binding pocket using the same docking parameters applied to the test ligands. The resulting binding poses were compared with the crystallographic orientation, and the calculated root-mean-square deviation (RMSD) values were all below 2.0 Å.^28–30^ This confirmed the reliability and accuracy of the docking setup, ensuring that the protocol could reproduce experimentally observed ligand–protein interactions with high fidelity.

#### 2.4.3 Interaction Analysis and Visualization of Ligand–Protein Complexes

Following molecular docking, the resulting ligand–protein complexes were imported into BIOVIA Discovery Studio Visualizer 2021 to identify and analyze key molecular interactions between CaONPs and target outer membrane proteins.^31^ The best-scoring docking poses (based on binding affinity values from AutoDock Vina) were selected for detailed interaction mapping. The Receptor–Ligand Interactions module was used within Discovery Studio. For each docked complex, the ligand (CaONP or reference compound) was defined using the “Define Ligand” tool, and interacting residues were visualized via the “Analyze Ligand Interactions” function. The interaction maps generated included as following: Hydrogen bonds (green dashed lines), Electrostatic interactions (orange lines), and Hydrophobic and π–π stacking interactions (red lines). Each interaction was automatically annotated with the corresponding amino acid residue and its position. These interactions were saved as high-resolution 2D diagrams for visual representation in the manuscript. All visualizations were prepared with default settings and adjusted manually to improve the clarity where necessary.

### 2.5 Antibacterial and Antifungal Activity Tests

Antibacterial and antifungal activities of the CaONPs were determined before and after calcination. Antimicrobial activities of the synthesized nanoparticles were tested using the agar well diffusion method. 25 μL of *E. coli* and *S. aureus* suspensions prepared according to the 0.5 McFarland turbidity standard (10^8^ CFU/mL) were inoculated into the medium. 25 μL of nanoparticle solution (1 mg/mL) was applied to the wells (5 mm in diameter) opened on the nutrient environment and incubated for 24 h at 37 °C. The zone diameters (mm) were measured at the end of the incubation period. Commercial streptomycin and ampicillin, known to have antimicrobial activity, were used as positive controls (25 μL). Antifungal susceptibility of the synthesized nanoparticles was assessed by an agar diffusion test. PDA medium was inoculated with 25 μL of *A. niger* and *C. albicans* (10^8^ CFU/mL) and 25 μL of 100 μg/mL CaONP solution, and incubated at 37 °C, and the zones formed after 24 h were measured. These zones were checked against the zones of antifungal antibiotics amphotericin B (80 μg/mL) and clotrimazole (50 μg/mL). Distilled water was used as a negative control in all experiments. Each experiment was performed with 3 replicates.

### 2.6 *In Vitro* Release Studies

The drug loading process was carried out by the adsorption of Zoledronic acid (ZA) onto nanoparticles. Nanoparticle solution at a concentration of 1 mg/mL was prepared using PBS at pH 7.4. It was mixed with 5 mg of ZA at room temperature for 2 h (ZA:CaONP 1:2 (w/w)). Then, the prepared solution was centrifuged at 10.000 rpm for 15 min. It was washed, and drug-loaded nanoparticles were dried. Drug loading capacity (LC) was determined by the following formula:

$$\mathrm{LC}=\frac{t\mathrm{otal}\;\mathrm{ZA}-f\mathrm{ree}\;\mathrm{ZA}}{\mathrm{CaONP}\;\mathrm{weight}}\times 100$$

Drug release studies were performed using sodium acetate buffer (pH 5.0) and phosphate buffer (pH 7.4). A 10 mg portion of ZA-loaded nanoparticle was taken into 10 mL of buffer. Drug release was performed in a slightly shaking water bath at 37 °C. Absorption was read at 220 nm, which is the absorption wavelength of ZA at certain time intervals (30 min, 1 h, 2 h, 4 h, 8 h, 24 h, 2 days, 3 days, 5 days, 7 days, and 10 days). The drug loading process is depicted in Scheme 2. The amount of ZA released from the nanoparticle was determined using the calibration curve obtained by the absorbance values of solutions with a concentration range of 0.02–0.6 mg ZA/mL PBS. Each experiment was performed with 3 replicates.

**Scheme 2:**
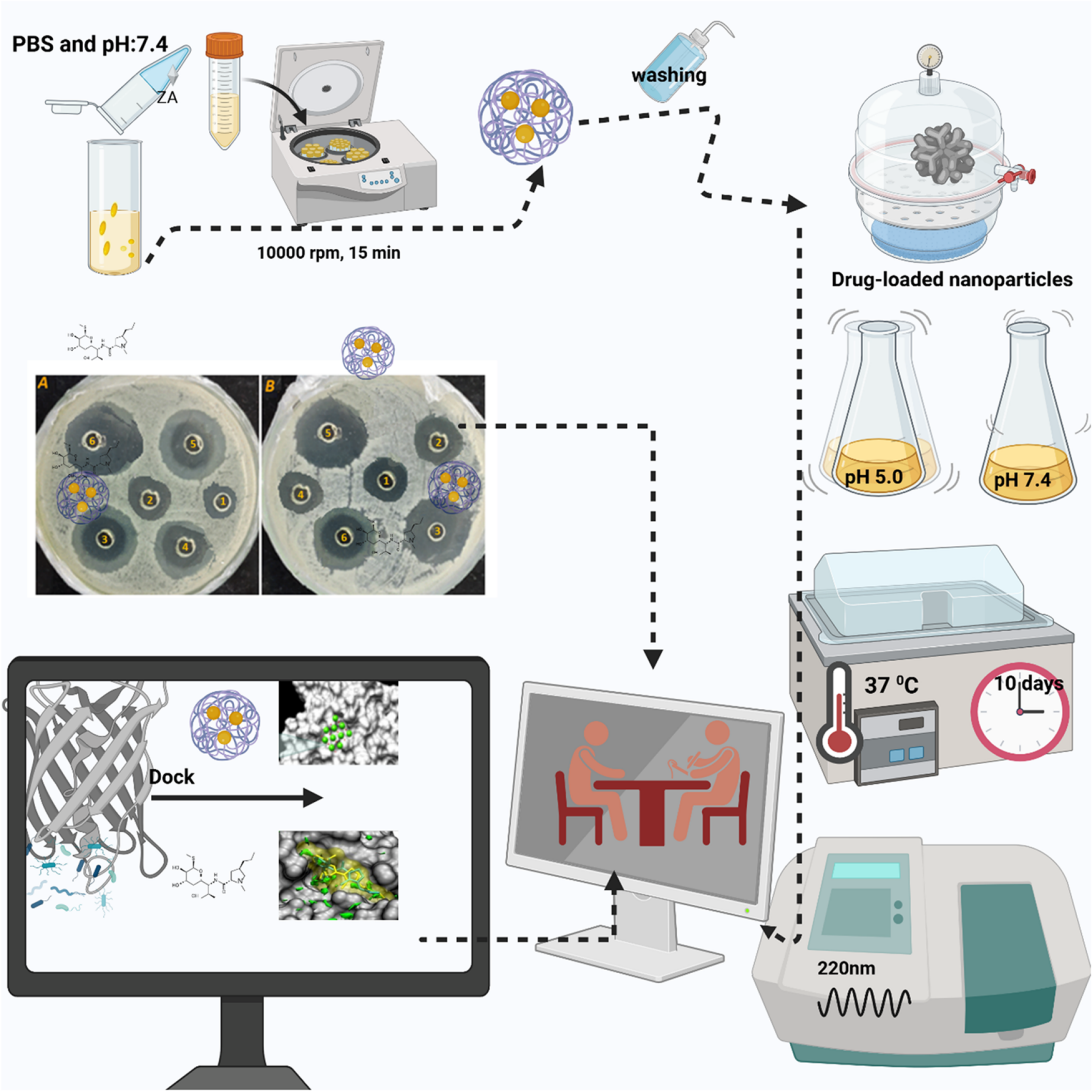
Overview of the Drug Loading Process of CaONPs

### 2.7 MTT Assay

In the MTT assay performed, the proportion of viable cells within the cell population was quantitatively measured by a microplate reader spectrophotometer. Saos-2 (HTB-85, ATCC, VA) human osteosarcoma (passage 14) cells were prepared as a suspension in McCoy’s 5A medium (supplemented with 15% fetal bovine serum and 1% penicillin/streptomycin) at a concentration of 1 × 10^4^ cells/well and seeded into 96-well culture plates. After the cells were incubated overnight at 37 °C in 5% CO_2_ conditions, six different concentrations of the test sample were prepared (CaONP concentrations: 1000–500–250–125–62.50–31.25 μg/mL) and applied in triplicate for 24 h. At the end of the exposure period, the medium in the wells was replaced with PBS, and 50 μL of MTT solution at a concentration of 0.5 mg/mL was added to each well. After the cells were incubated at 37 °C in a CO_2_ incubator for 3 h, the formazan crystals formed were dissolved in dimethyl sulfoxide (DMSO). Absorbance was measured using a spectrophotometer at 570 nm. Concentrations with three biological replicates and a control group were tested. Results were evaluated as the percentage of cell viability compared to the control group.

### 2.8 Dye Degradation Analysis

The dye removal effect of CaONPs using Congo red was investigated. 20 mg of CaONPs was weighed and mixed with 5 mL of Congo red (0.1 mg/mL). The absorbance values of this mixture between 10 min and 24 h were measured by UV–vis spectroscopy under two different environmental conditions. In the first stage, the temperature was kept constant (25 °C), and the pH was studied at 5.0 and 9.0. In the second stage, the temperature parameter was investigated by keeping the pH constant at 5.0, and the experiments were carried out at 25 and 50 °C. Each experiment was performed with 3 replicates. The calculation of the percentage decolorization of the CaONPs was performed by measuring the decrease in absorbance at the specific λ_max_ of Congo red over a 90 min period using a UV–vis spectrophotometer (model UV-1700 Pharmaspec Shimadzu). The following formula, which explains dye removal efficiency (DE), absorbance of the dye (Ci), and absorbance of the dye after interaction with the CaONPs (Cf), was used to calculate the percentage of dye degradation:

$$\%\;\mathrm{DE}=\frac{\mathrm{Ci}-\mathrm{Cf}}{\mathrm{Ci}}\times 100$$

### 2.9 Statistical Analysis

The results obtained from the tests concerning membrane integrity and cell viability were analyzed using an ordinary one-way ANOVA test, complemented by Tukey’s multiple comparisons of means. When the *p* value fell below 0.05, the means were considered significantly different. One-way ANOVA was utilized for statistical analysis with a confidence level of 95%.

## 3 Results and Discussion

### 3.1 Synthesis and UV–vis Analyses of CaONPs

The CaONPs synthesized via *S. japonica* were analyzed by UV–vis spectroscopy before and after the calcination process (Figure 1). The *S. japonica* extract was also analyzed by UV–vis and exhibited strong absorption peaks at 259 and 332 nm. These peaks can be attributed to the presence of polyphenols, flavonoids, and other biomolecules that are naturally found in the plants. The peaks suggested the presence of chromophores, likely due to π–π* and n–π* electronic transitions in aromatic rings and conjugated systems. The absorption spectrum of CaONP I showed significant peaks at 272 and 321 nm. This suggested that biomolecules from the plant extract may have interacted with calcium oxide nanoparticles, thus influencing the absorbance pattern. The broader absorption band in the UV region indicated the presence of interactions between biomolecules and nanoparticles, which were possibly stabilizing the CaONPs. After calcination, the spectrum of CaONP II showed a significant reduction in the absorbance. It presented a gradual decrease in absorbance from 200 to 500 nm, indicating that pure CaONPs did not exhibit strong UV absorption. The broad and weak absorbance peak suggested the removal of organic biomolecules during the calcination process that was applied at 500 °C. The reasons for this significant decrease in the absorbance may be related to the changes in the structure of the nanoparticles, an increase in the size of the nanoparticles, or changes in their chemical composition. Calcination at 500 °C may change the crystal structure of nanoparticles produced from plant extracts. This may cause the nanoparticles to lose their UV-absorption properties, or the nanoparticles may coalesce or sinter during calcination, which may cause the particle size to increase. Larger particles may lose their UV-absorption properties. Alternatively, organic compounds in the plant extract may burn or evaporate during calcination. This may change the chemical composition of the nanoparticles and thus their UV-absorption properties. The presence of plant extract in the synthesis phase contributed to the initial strong absorbance due to biomolecular interactions with CaONP I. The transformation from CaONP I to CaONP II was evident in the spectral change, confirming the formation of CaONPs with minimal organic contamination. The UV–vis spectra successfully confirmed the transition from CaONP I (with organic stabilizers) to CaONP II (with a pure inorganic phase). The plant extract played a key role in the biosynthesis process, acting as a reducing and stabilizing agent. The UV–visible absorption spectra obtained from the biosynthesis of CaONPs using *S. japonica* fruit extract align well with findings in recent literature. In a study, CaONPs synthesized using *Moringa oleifera* leaf extract exhibited an absorption peak at approximately 323 nm, which was attributed to the presence of polyphenolic compounds acting as reducing agents during nanoparticle formation.^32^ In another report, CaONPs synthesized via the direct precipitation technique exhibited a CaO peak at 360 nm in UV–vis analysis.^33^ These studies, along with others, demonstrated that plant extracts rich in polyphenols and flavonoids can effectively facilitate the synthesis of CaONPs, resulting in characteristic UV–vis absorption peaks. The observed spectral features in CaONPs I and II are consistent with these findings, confirming the successful synthesis of CaONPs by using *S. japonica* fruit extract.

**Figure 1:**
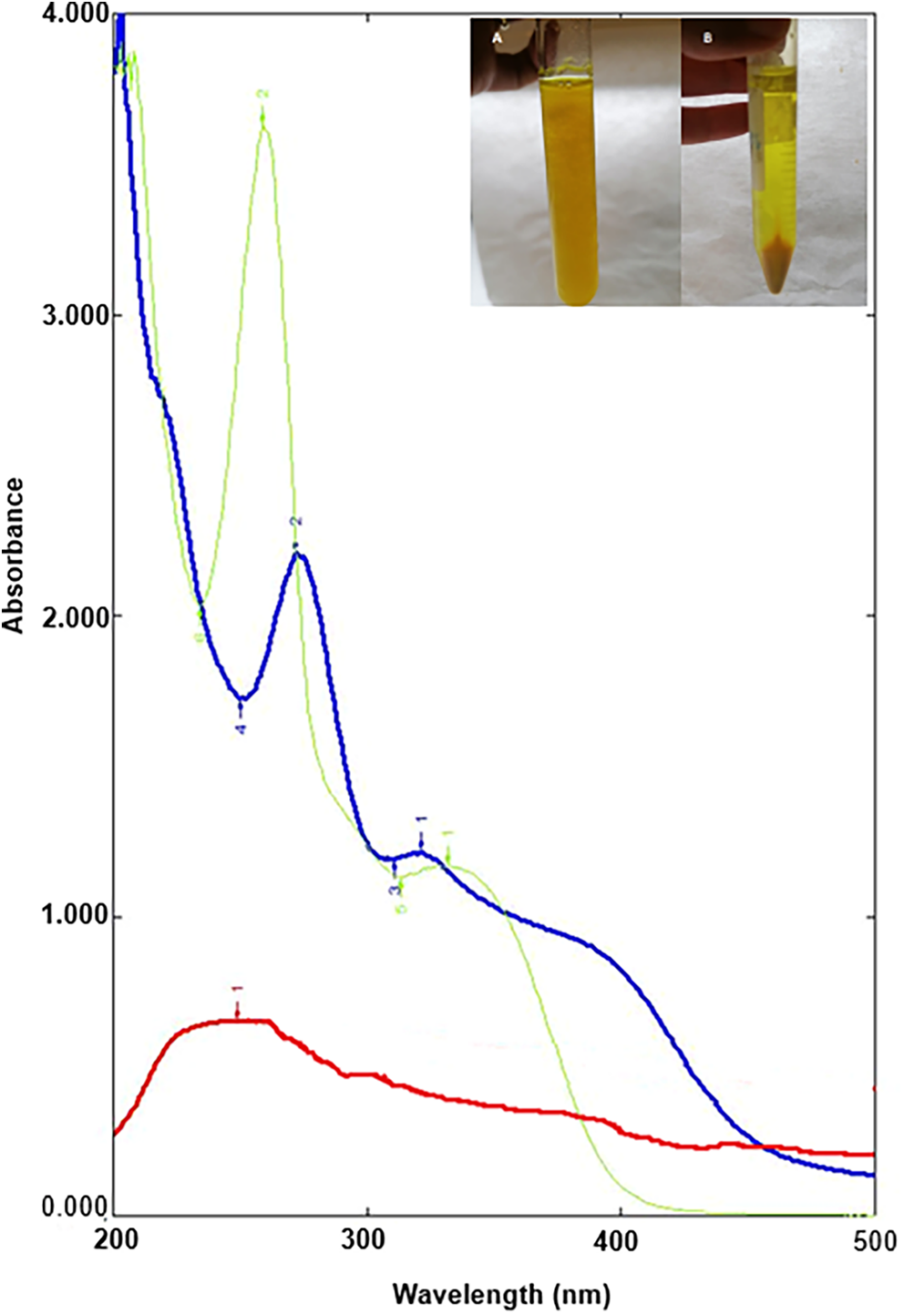
UV spectra of *S. japonica* extract (green), CaONP I (blue), and CaONP II (red); and the images of nanoparticle synthesis before (A) and after (B).

### 3.2 Characterization of CaONPs

The FT-IR analysis of the CaONPs was applied before and after calcination, providing detailed information about the transformation of the nanoparticle and the removal of organic components during the heat treatment process (Figure 2). Figure 2a represents the peaks obtained by the *S. japonica* fruit extract, including the wavelength numbers 3272, 2987, 2900, 1607, 1506, 1408, 1241, 1065, and 834 cm^–1^. The FT-IR spectrum of CaONP I (before calcination) exhibited peaks at 3644, 2987, 2900, 2160, 1612, 1393, 1251, 1227, 1065, and 867 cm^–1^ (Figure 2b). The broad peak at 3644 cm^–1^ corresponded to the O–H stretching vibration, indicating the presence of hydroxyl groups originating from residual water or plant-derived organic compounds. The peaks at 2987 and 2900 cm^–1^ were associated with C–H stretching vibrations, confirming the presence of organic residues such as proteins, flavonoids, or phenolic compounds originating from the S. japonica extract. The peak at 2160 cm^–1^ may be attributed to C≡C or C≡N stretching, which was indicative of alkyne or nitrile functional groups. This 2160 cm^–1^ peak suggested an interaction between organic compounds and CaONPs. The peak at 1612 cm^–1^ was characteristic of C=O stretching of carbonyl groups and can also be linked to amide or conjugated C=C bonds, traceable to phytochemicals acting as capping agents. The band at 1393 cm^–1^ represented carbonate (CO_3_^–2^) vibrations, suggesting that the precursor material contained some degree of carbonate species. Peaks at 1251 and 1227 cm^–1^ were attributed to C–O stretching vibrations, which can be associated with alcohols, carboxyl, or phenolic groups present in the plant extract. The strong peak at 1065 cm^–1^ corresponded to C–O–C stretching; this may indicate the presence of polysaccharides or esters in the organic matrix of the fruit extract. The peak at 867 cm^–1^ suggested the presence of Ca–O bonds, demonstrating the formation of calcium-based compounds.

**Figure 2:**
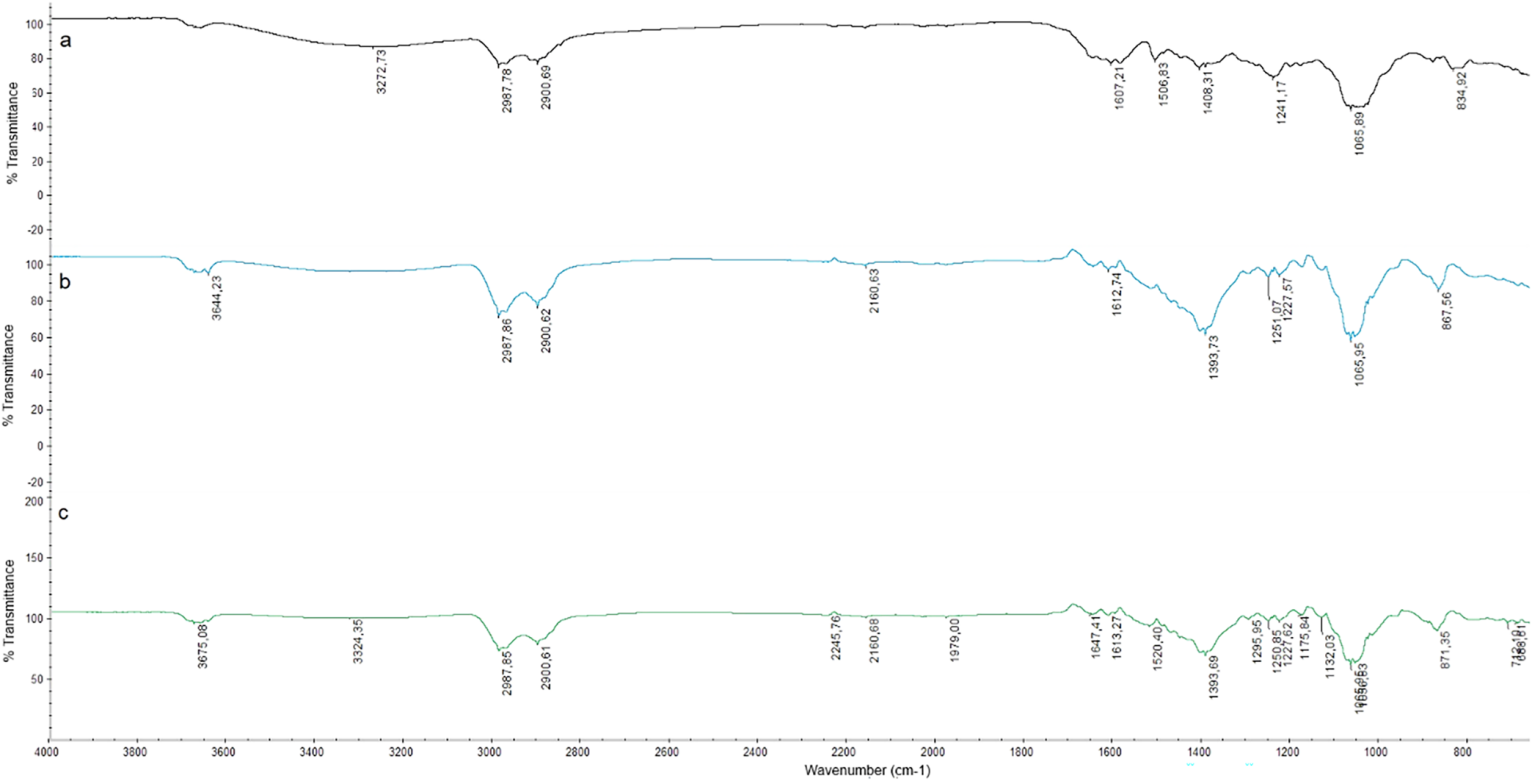
FT-IR spectra of (a) *S. japonica* extract, (b) CaONP I, and (c) CaONP II.

The FT-IR spectroscopy of CaONP II (after calcination) revealed peaks at 3675, 3324, 2987, 2900, 2245, 2160, 1979, 1647, 1613, 1520, 1393, 1295, 1250, 1227, 1175, 1132, 1065, 1056, and 871 cm^–1^. These peaks indicated the presence of various functional groups associated with the CaO structure (Figure 2c). The broad peak at 3675 cm^–1^ corresponded to the O–H stretching vibration, suggesting surface hydroxyl groups contributed to the hydrophilic nature of the nanoparticles. The peak at 3324 cm^–1^ can also be attributed to hydrogen-bonded hydroxyl (−OH) groups, possibly from residual plant-derived organic compounds. The bands at 2987 and 2900 cm^–1^ indicated the presence of organic residues from phytochemicals used in the green synthesis process and corresponded to the C–H stretching vibrations of aliphatic hydrocarbons. The peaks at 2245 and 2160 cm^–1^ were assigned to C≡C or C≡N stretching vibrations, which may have arisen from the plant metabolites interacting with the nanoparticle surface. The peaks at 1647 and 1613 cm^–1^ were characteristic of C=O and C=C stretching vibrations and are often linked to amide or aromatic compounds. The peak at 1520 cm^–1^ may indicate aromatic C=C stretching, further supporting the involvement of plant-derived compounds in nanoparticle stabilization. The absorption bands at 1393 cm^–1^ corresponded to symmetric stretching vibrations of carbonate (CO_3_^–2^), suggesting slight carbonation of the CaONPs due to atmospheric CO_2_ exposure. The peaks at 1295, 1250, 1227, and 1175 cm^–1^ can be assigned to C–O stretching vibrations originating from phenolic or carboxyl functional groups. The presence of peaks at 1132, 1065, and 1056 cm^–1^ corresponded to C–O–C or sulfate-related vibrations, which may be attributed to residual bio-organic components. Lastly, the sharp band at 871 cm^–1^ was a well-known characteristic peak of Ca–O stretching vibrations; thus confirming the formation of CaONPs. These results are parallel with a previous report on the green synthesis of CaONPs via *Rhododendron arboreum*.^34^ As reported in the literature, Ca–O vibrations typically occur between 400 and 900 cm^–1^.^32,35,36^

As seen from the results, significant changes in the FT-IR spectrum were observed, which are signs of the removal of organic matter and the formation of pure CaO. The disappearance or reduction of peaks related to C–H (2987 and 2900 cm^–1^), C=O (1612 cm^–1^), and C–O–C (1065 cm^–1^) confirmed the degradation and/or elimination of organic residues due to high-temperature treatment. The intensity of the carbonate-related peak (1393 cm^–1^) remained, implying a partial interaction with atmospheric CO_2_ and leading to minor carbonation of the CaONPs. Additionally, new peaks such as 3675, 3324, 1979, 1647, 1520, and 1132 cm^–1^ appeared in the spectra because of some structural modifications or hydroxylation or atmospheric adsorption effects. Overall, the FT-IR analysis before and after calcination confirmed the successful synthesis of CaONPs with additional functional groups, pointing out the presence of plant-derived compounds acting as capping or stabilizing agents. Before calcination, the presence of hydroxyl (−OH), carbonyl (C=O), and organic (C–H, C–O–C) functional groups suggested strong interactions with phytochemicals derived from S. japonica. After calcination, these peaks were significantly reduced or disappeared and thereby confirmed the removal of organic material and the formation of crystalline CaONPs. This transformation was crucial for ensuring the purity and stability of the synthesized CaONPs for potential applications in catalysis, the biomedical fields, and environmental remediation. Importantly, this study contributes novel insights by documenting intermediate spectral features, which suggest more complex plant–nanoparticle interactions than previously detailed. These peaks point to a richer variety of phytochemical capping agents involved in the stabilization of CaONPs synthesized from *S. japonica*, which have not been addressed in earlier reports.

The morphological characteristics and particle size distribution of the synthesized CaONPs were investigated by using SEM analysis to gain insight into their surface structure and nanometric features, which are crucial for understanding their functional properties. The SEM image of the CaONPs obtained after the calcination process (CaONP II) revealed critical morphological characteristics of the synthesized nanoparticles (Figure 3). The micrograph showed that CaONPs exhibited a predominantly spherical and agglomerated morphology. The particles appear to form large and porous clusters that may have resulted from strong interparticle interactions due to high surface energy. The surface of the agglomerates, which can be seen at higher magnification, consists of numerous nanosized particles, indicating a rough and porous texture. This structure is typical for CaONPs synthesized via green routes because the removal of organic components during calcination can lead to porosity. However, the high porosity observed in Figure 3 may enhance the surface area by making the CaONPs potentially useful in applications such as catalysis, adsorption, and biomedicine. The uniform particle distribution suggested that the calcination process effectively eliminated organic residues and led to well-defined nanostructures. Also, the CaONPs exhibited a hierarchical structure with small primary nanoparticles aggregated to form larger spherical clusters. The primary nanoparticle sizes appeared to be in the range between 30 and 70 nm, while the larger agglomerates reached the micrometer scale (approximately 1–2 μm in diameter). This aggregation behavior was commonly observed in CaONPs synthesized via green methods. Plant-derived organic compounds initially act as stabilizing agents but decompose during calcination, thus leading to clustering. Despite the agglomeration, the nature of CaO was evident from the fine and granular texture observed at a high magnification. The SEM analysis of the CaONPs synthesized using *S. japonica* fruit extract revealed a predominantly spherical morphology with a tendency to form agglomerates. This morphological consistency in plant-mediated syntheses highlights the influence of natural extracts on the formation and stabilization of CaONPs. These findings are consistent with previous studies, such as those by Huda et al. (2022) and Bano and Pillai (2020), who also reported similar morphologies and size distributions for CaONPs synthesized via green routes using *Citrullus colocynthis* and *Murraya koenigii* extracts, respectively.^36,37^ However, compared to those reports, the particles in the present study exhibited more uniform morphology with a relatively porous surface texture, likely due to the unique composition of phytochemicals present in *S. japonica*, which may act as effective capping and stabilizing agents. Furthermore, the moderate degree of agglomeration observed is in agreement with a finding, which emphasized that postcalcination treatments often enhance particle clustering.^37^ Overall, the SEM results confirm the successful synthesis of well-defined CaONPs and suggest that *S. japonica* extract contributes favorably to controlling particle morphology, offering potential advantages in biomedical and catalytic applications where surface uniformity and particle stability are critical.

**Figure 3:**
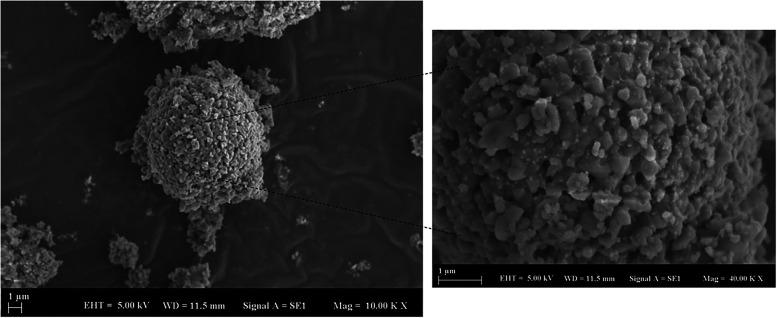
SEM images of the CaONPs obtained after the calcination process (CaONP II).

The zeta potential analysis of the synthesized CaONP II revealed a value of −15.7 ± 3.17 mV (Figure 4A), suggesting that the nanoparticles exhibit moderate colloidal stability in an aqueous suspension. Typically, nanoparticles with zeta potentials greater than ±30 mV are considered highly stable due to strong repulsive forces, whereas those in the range of ±10 to ±30 mV show moderate stability with a tendency toward aggregation under certain conditions.^38^ The single, symmetrical peak in the zeta potential distribution confirmed the uniformity of the nanoparticle population and the absence of multiple particle species or significant aggregation. Comparable zeta potential values have been reported for CaONPs synthesized via other green approaches. For instance, Eram et al. (2021) reported a zeta potential of −23 ± 11 mV for CaONPs synthesized using *Crescentia cujete* leaf extract, while Sharma et al. (2023) noted a value of −21.6 mV when using *Cleome viscosa* leaf extract, both indicating moderate electrostatic stabilization.^39,40^ The slightly lower zeta potential in this study may be attributed to the specific phytochemical composition of *S. japonica*, which likely acts as a capping agent through hydrogen bonding or weak electrostatic interactions. Biomolecules such as polyphenols, proteins, and polysaccharides are known to adsorb onto nanoparticle surfaces, modulating the surface charge and influencing colloidal behavior.^41^ Therefore, the moderate zeta potential observed here supports the conclusion that *S. japonica* contributes to particle stabilization, although not to the same extent as the case for stronger ionic or polymeric surfactants. This surface charge behavior is consistent with our SEM findings, which showed moderate agglomeration likely governed by a balance between van der Waals attractions and residual repulsive forces.

**Figure 4:**
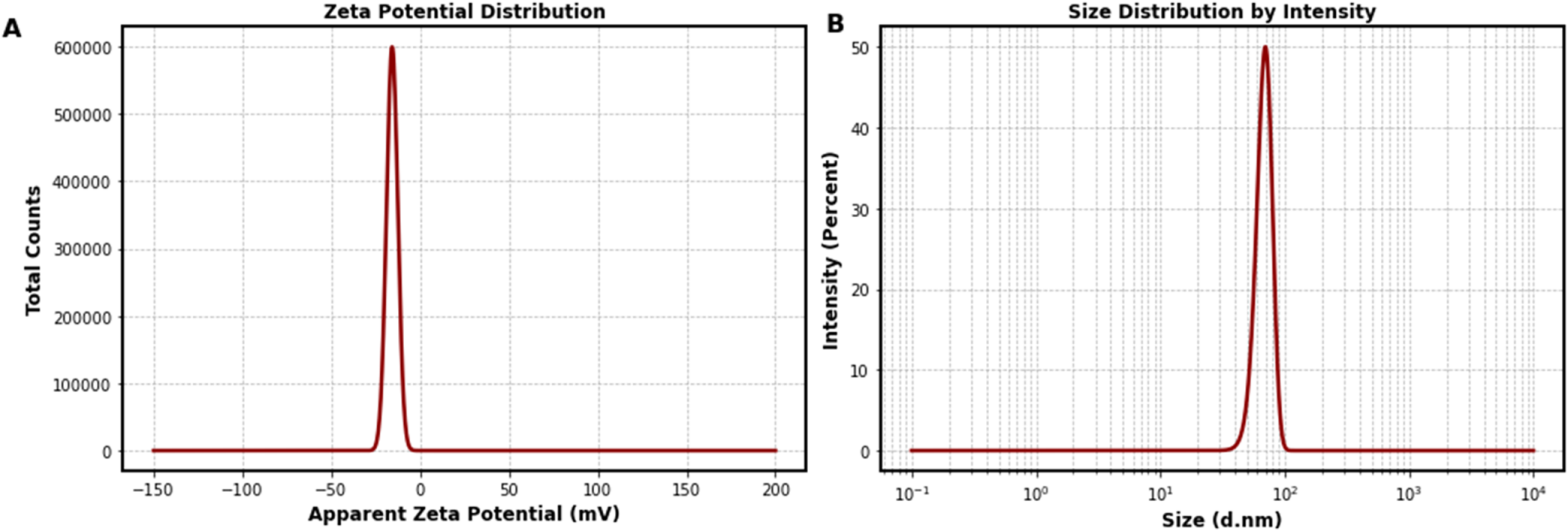
Ζ-potential (A) and size distribution (B) of the CaONPs obtained after the calcination process (CaONP II).

The DLS analysis of CaONPs indicated a size distribution with a peak at 72 nm, suggesting a comprehensible hydrodynamic diameter compared with the SEM results. The DLS graph showed a sharp and narrow peak, indicating a monodisperse distribution of nanoparticles with minimal aggregation (Figure 4B). The size distribution was centered around 60–80 nm, suggesting that the synthesized CaONPs were in the nanoscale regime. SEM showed CaONPs in the range of 30–70 nm, while DLS reported sizes around 60–80 nm because of the hydrodynamic radius effect in DLS. Both DLS and SEM confirmed that the CaONPs were in the nanoscale range with good dispersity. A previous study on green-synthesized CaONPs reported similar sizes (60–70 nm) utilizing the leaf extract of *Ocimum tenuiflorum*.^42^ The high intensity peak at a single size range confirmed the uniformity of the CaONPs, which is essential for a consistent biological and catalytic performance. Moreover, the absence of broad or multiple peaks suggested good dispersion stability in the solvent medium, making the synthesized nanoparticles suitable for various biomedical and environmental applications.

The X-ray diffraction analysis of the CaONPs synthesized via *S. japonica* extract confirmed the successful formation of crystalline calcium oxide. The diffraction peaks observed at 29.29, 35.89, 39.31, 43.07, 47.37, and 48.39° (2θ) corresponded to the characteristic reflections of CaO, matching well with the standard JCPDS No. 37–1497 (Figure 5). The most intense peak observed at 29.29° (2θ) with the highest relative intensity (100%), which is a characteristic peak of CaO, indicates the dominant (111) crystal plane with a highly ordered crystalline structure. The presence of additional minor peaks at lower angles might indicate residual traces of Ca­(OH)_2_ or CaCO_3_, which are commonly formed due to atmospheric moisture or CO_2_ absorption. The relatively sharp and intense peaks along with narrow full width at half-maximum (fwhm) values between 0.1279 and 0.2047° suggested a high degree of crystallinity in the synthesized nanoparticles. Similar peak positions were reported with slight variations due to differences in synthesis methods and calcination temperatures of CaONPs synthesized using *M. koenigii* leaf extract.^37^ To determine the crystallite size, the Scherrer equation was applied to the most intense peak, and the calculated crystallite size of the CaONPs was 53.45 nm. Another report on the green synthesis of CaONPs has similarly demonstrated high crystallinity with diffraction patterns at 29.61° (011), 32.17° (111), 37.27° (200), and 54.26° (022), corresponding to CaO, depicting the preferred cubic crystalline nature and average size of 32.12 nm.^43^ However, in contrast to some reports that indicated broader peaks due to lower crystallinity or incomplete calcination, the narrow fwhm values observed in this study support the superior crystalline nature of the nanoparticles produced by using *S. japonica*. Moreover, the crystallite size (53.45 nm) falls within the typical range (20–60 nm) reported for biogenic CaONPs, further validating the effectiveness of the phytochemical-rich *S. japonica* extract in producing high-quality crystalline nanoparticles. These findings demonstrated that *S. japonica* extract effectively facilitated the synthesis of well-crystallized CaONPs. Having confirmed the successful synthesis and physicochemical features of CaONPs, the study proceeded to evaluate their potential biological interactions through molecular docking analyses targeting key microbial and therapeutic proteins.

**Figure 5:**
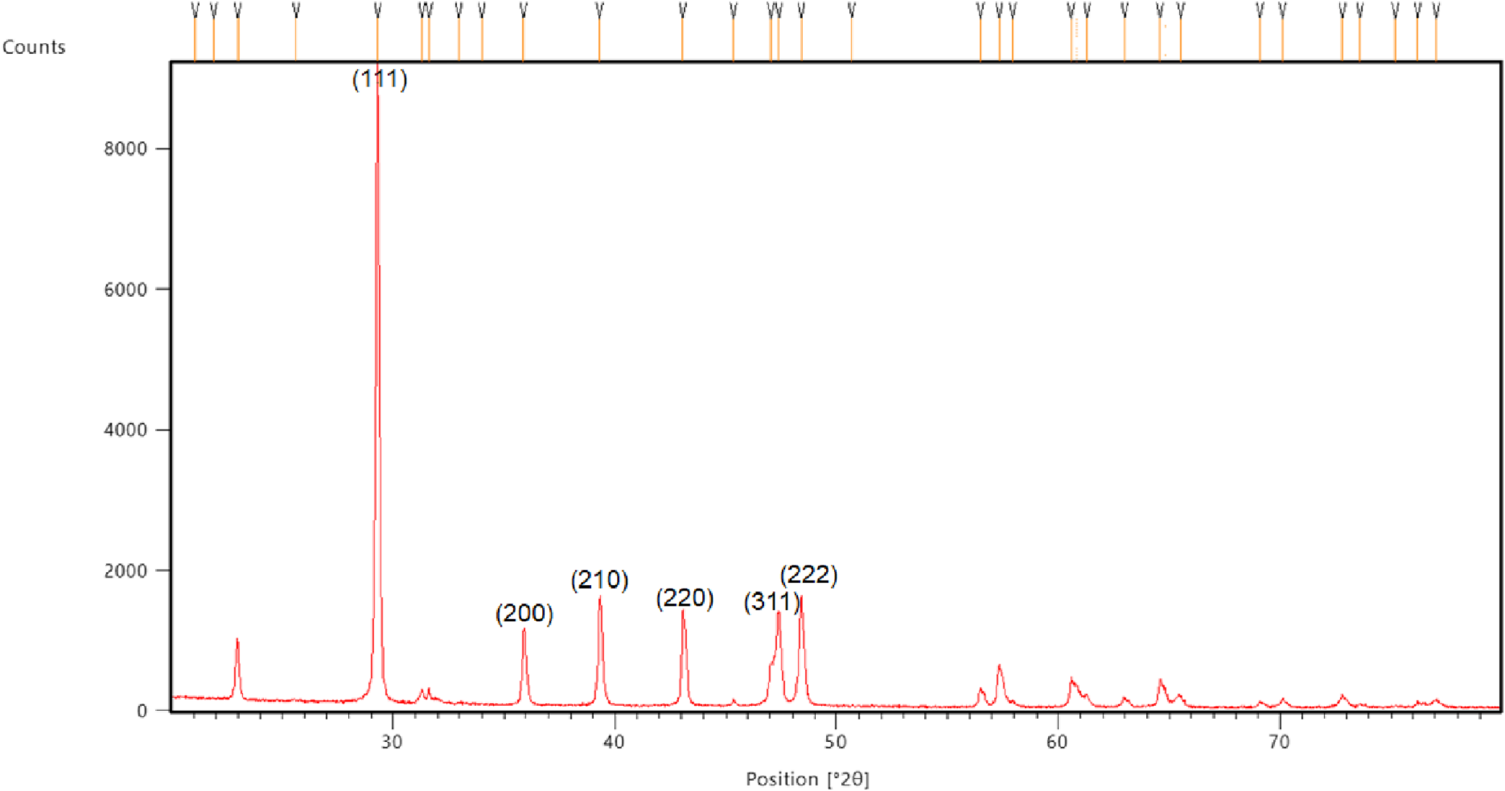
XRD spectrum of CaONPs synthesized via *S. japonica*.

### 3.3 Molecular Docking Analyses

The molecular docking analysis revealed favorable interactions between the synthesized CaONPs and Zoledronic acid with the Farnesyl diphosphate synthase enzyme (PDB ID: 1ZW5). Panel A shows that the CaONP was successfully docked into the enzyme’s active pocket, achieving the best binding energy score of −7.0 kcal/mol, indicating a strong and stable interaction (Figure 6). Panel B represents the docking of Zoledronic acid, which is also located within the binding pocket and exhibits a slightly lower binding affinity of −6.7 kcal/mol. These results suggest that CaONP demonstrates comparable or even slightly enhanced binding efficiency to the target protein when compared to the reference drug, Zoledronic acid.

**Figure 6:**
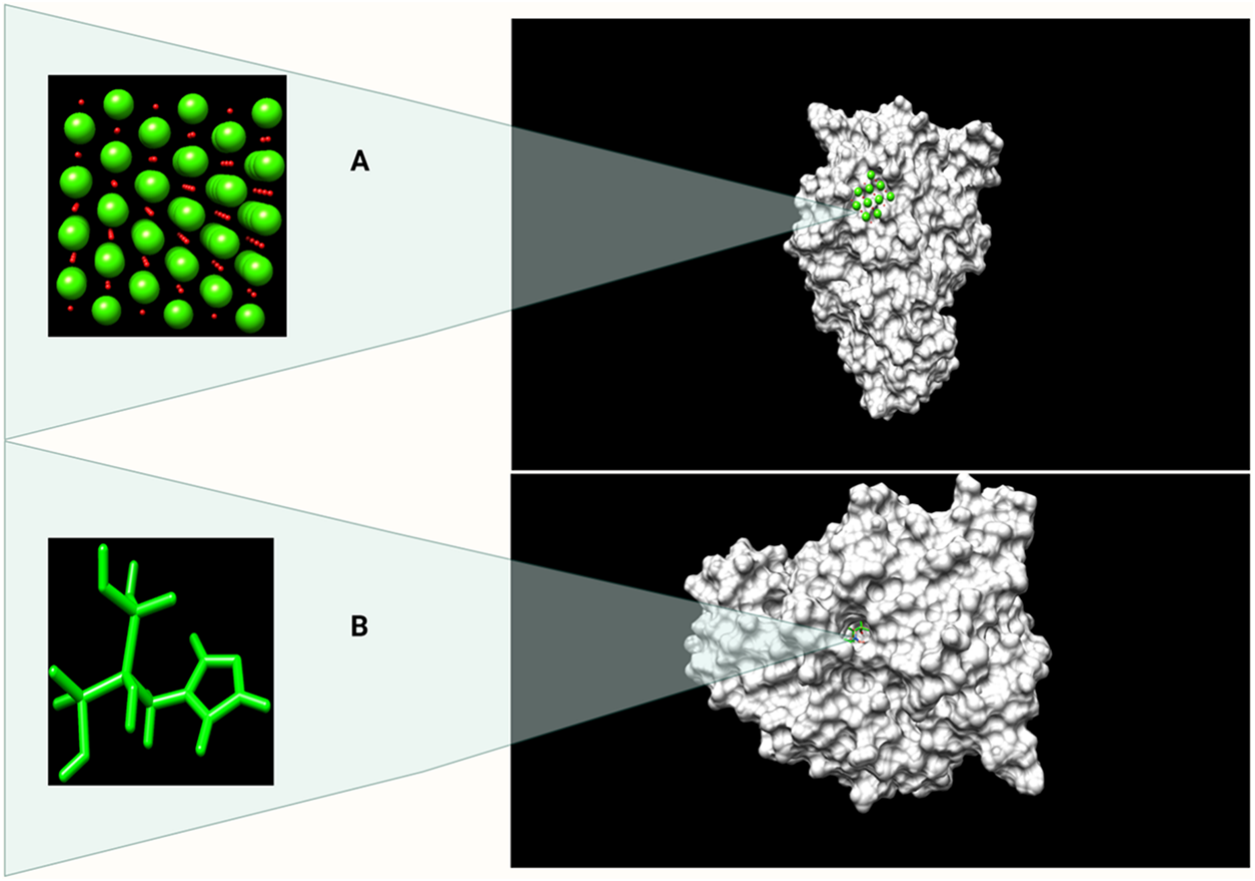
Molecular docking analysis showing the interaction between CaONPs (green and red spheres) and Zoledronic acid (stick representation) with Farnesyl diphosphate synthase protein (PDB ID: 1ZW5). Panel (A) illustrates the docking of CaONPs into the active site of the protein, while Panel (B) shows the interaction of Zoledronic acid.

Based on the molecular docking analyses, the binding affinities of the CaONPs against selected outer membrane proteins were found to be comparable to or higher than those of known reference compounds (Table 1). For the 1FEB protein, CaO exhibited a binding score of −9.9 kcal/mol, which is stronger than those of CCCP (−7.4 kcal/mol) and our nanoparticle. The lowest binding energy for this protein was observed with Cefiderocol (−10.5 kcal/mol), while the highest binding energy was observed with Gallium maltolate (−5.8 kcal/mol). Similarly, for the 1BY3 structure, CaO showed a binding affinity of −9.4 kcal/mol, surpassing those of CCCP (−6.5 kcal/mol) and most other compounds. The most favorable interaction for this protein was observed with Dacrobactin (−9.8 kcal/mol), and the weakest interaction was observed with Gallium maltolate (−5.6 kcal/mol). In the case of 4C4V, CaO demonstrated a binding score of −9.5 kcal/mol, which is higher than those of CCCP (−6.2 kcal/mol) and Cefiderocol (−8.7 kcal/mol). The strongest binding was again with Dacrobactin (−9.8 kcal/mol) and the weakest with Gallium maltolate (−5.5 kcal/mol). For the 2HDF protein, CaO’s binding affinity was −9.2 kcal/mol, exceeding CCCP (−6.7 kcal/mol) and being comparable to Cefiderocol (−9.1 kcal/mol) and Dacrobactin (−9.6 kcal/mol). The lowest energy was shown by Dacrobactin (−9.6 kcal/mol) and the highest by Gallium maltolate (−5.5 kcal/mol). Regarding the 7NG9 protein, CaO achieved a score of −8.8 kcal/mol, which was close to that of Dacrobactin (−8.6 kcal/mol) and significantly higher than that of CCCP (−6.5 kcal/mol). For this protein, CaO itself (−8.8 kcal/mol) showed the lowest binding energy, while the weakest interaction occurred with Gallium maltolate (−5.2 kcal/mol). And, the Figure 7 illustrates the predicted binding poses of synthesized CaONPs within the binding cavities or surface regions of five distinct outer membrane proteins: 7NG9, 1BY3, 1FEB, 2HDF, and 4C4V. Each panel represents a separate docking simulation in which CaO is positioned within the structurally accessible regions of the target proteins (rendered in ribbon style).

**Figure 7:**
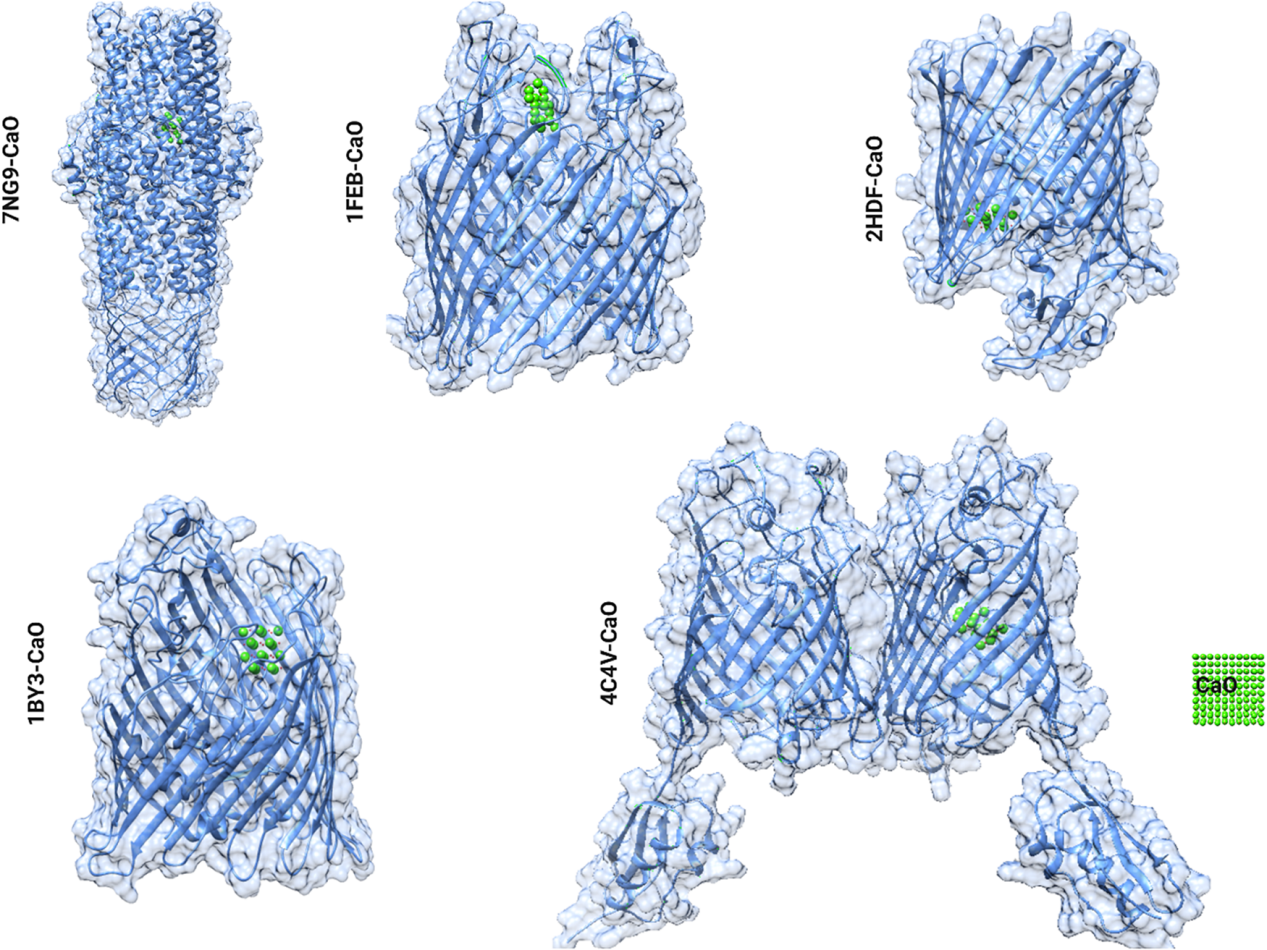
Molecular docking analysis showing the interaction between CaONPs (green spheres) and selected bacterial outer membrane proteins.

**Table 1:** Molecular Docking-Derived Binding Affinities (kcal/mol) of Selected Antimicrobial Compounds and CaONP against Bacterial Outer Membrane Proteins^a^

**compounds**	Colistin	Cefiderocol	Dacrobactin	Gallium maltolate	CCCP	CaO
**protein**						
1FEB	–7.1	–10.5	–10.1	–5.8	–7.4	–9.9
2HDF	–7.6	–9.1	–9.6	–5.5	–6.7	–9.2
1BY3	–7.4	–8.9	–9.8	–5.6	–6.5	–9.4
4C4V	–8.2	–8.7	–9.8	–5.5	–6.2	–9.5
7NG9	–7.8	–8.6	–8.6	–5.2	–6.5	–8.8

a CCCP: Carbonyl Cyanide m-Chlorophenyl Hydrazone, CaO: Calcium Oxide.

The *in silico* docking analyses conducted in this study aimed to assess the binding potential of synthesized CaONPs in comparison with clinically relevant compounds targeting both eukaryotic and prokaryotic (outer membrane) proteins. The findings revealed promising interaction characteristics of CaONPs, suggesting their potential utility as novel bioactive agents. Initial docking simulations focused on Farnesyl diphosphate synthase (FDPS), the validated molecular target of Zoledronic acid, widely used in osteoclast inhibition.^44^ Both CaO and Zoledronic acid successfully docked within the enzyme’s active site, and the nanoparticle demonstrated a slightly more favorable interaction (Figure 6 and Table 1). These results support the notion that CaO may act as a functional analogue or complementary agent to bisphosphonate-based drugs in bone-targeted therapies.

The comparative docking analyses with bacterial outer membrane proteins revealed that CaONPs exhibit broad and competitive binding behaviors (Table 1). In several cases, CaO showed binding affinities comparable to or greater than those of known antibiotics and metal-based inhibitors, including Colistin, Cefiderocol, Darobactin, and CCCP.^23–27,45^ These findings suggest that CaO may interfere with membrane-associated processes or protein functions critical for bacterial survival, thereby highlighting its potential as an antimicrobial agent. As depicted in Figure 8, CaONPs form stable hydrogen bonds and electrostatic interactions with critical amino acid residues such as GLU81, TYR558, ASP117, LYS1445, GLU404, ARG221, and ASP507. These residues are often located in functionally important regions of the outer membrane proteins, further supporting the proposed inhibitory mechanism of action. Also, these residues likely contribute to the stability and specificity of CaONP–protein interactions, further supporting their potential inhibitory effects.^46–48^ Importantly, the consistent performance of CaO across a diverse panel of OMPs underscores its multitarget interaction potential, which is especially valuable in combating bacterial resistance mechanisms. The observed binding trends suggest a membrane-targeting or pore-blocking mechanism, which could disrupt essential protein functions or compromise membrane integrity. Collectively, these results provide a strong rationale for the continued exploration of CaONPs as dual-action agents capable of engaging both human and bacterial targets. Such multifunctional behavior positions CaO as a promising candidate for further experimental evaluation in antimicrobial and bone-targeted therapeutic contexts.

**Figure 8:**
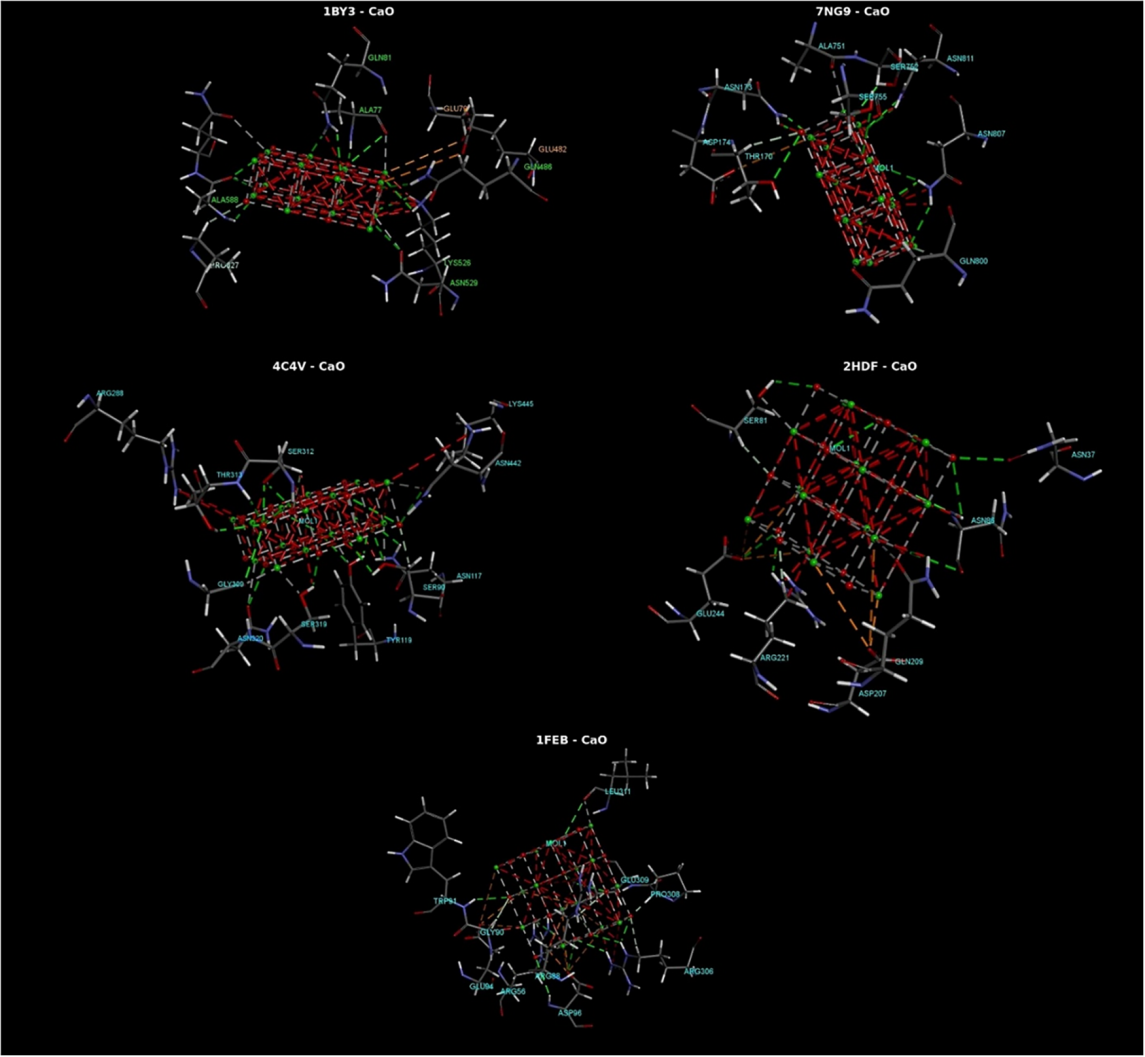
Detailed molecular interaction maps between CaONPs and the binding sites of selected outer membrane proteins (1BY3, 7NG9, 4C4V, 2HDF, and 1FEB).

As further supported by previous studies, metal oxide nanoparticles such as ZnO, TiO_2_, and Ga_2_O_3_ have been shown to exert antimicrobial effects by interacting with functional residues on outer membrane proteins, disrupting membrane integrity or enzyme function.^49–53^ Similar to these materials, *S. japonica*-mediated CaONPs formed strong interactions with residues such as GLU81, GLU482, and ASP507, which are involved in electrostatic and catalytic activities within the protein domains (Figure 8). These interactions suggest a possible mechanism of action, whereby CaONPs could inhibit transport or structural stability in bacterial membrane proteins. The comparative binding energy results and site-specific interactions thus highlight the dual functionality of CaONPs as both antimicrobial and osteotropic agents. Based on the molecular docking simulations suggesting strong interactions with bacterial outer membrane proteins, *in vitro* antimicrobial assays were performed to validate the predicted bioactivity of CaONPs against selected pathogens.

### 3.4 Antibacterial and Antifungal Activities of the CaONPs

The antibacterial activity of CaONPs was evaluated against *E. coli* and *S. aureus* by measuring the zone of inhibition (Figure 9). Before calcination, CaONP I exhibited moderate antibacterial activity, with inhibition zones of 12 mm for *E. coli* and 15 mm for *S. aureus*. After calcination, the inhibition zones increased to 16 mm for *E. coli* and 18 mm for *S. aureus*, suggesting enhanced antibacterial activity due to improved crystallinity, purity, or surface reactivity of the nanoparticles. Streptomycin and Ampicillin, used as positive controls, showed higher antibacterial activity than the nanoparticles alone. Streptomycin resulted in 19 mm inhibition for *E. coli* and 21 mm for *S. aureus*, *while Ampicillin had 18 mm* *E. coli* but only 14 mm for *S. aureus* (Table 2). However, a synergistic effect of antibiotics increased when combined with CaONPs. A significant increase in antibacterial effectiveness was obtained with the combination of the drug and CaONP II. Streptomycin and CaONP II resulted in 22 mm (*E. coli*) and 24 mm (*S. aureus*), an improvement over Streptomycin alone. Ampicillin and CaONP II led to 24 mm (*E. coli*) and 19 mm (*S. aureus*), showing a notable enhancement in the antibacterial action. The antibacterial activity results of CaONPs synthesized via *S. japonica* extract were found to be similar to a previous study on CaONPs synthesized using chicken eggshells, with the increasing inhibition zones of 17 ± 0.2 mm against *E. coli* and 19 ± 0.1 mm against *S. aureus* after calcination.^54^ There are studies in the literature comparing the effects of CaONPs on bacteria, such as *E. coli* and *S. aureus*, with some commonly used antibiotics. For instance, Awaad et al. (2023) reported antimicrobial activity of CaONP synthesized from Ca­(OH)_2_ precursor and compared their effectiveness to widely used antibacterial agents, including Amoxicillin, Cefoperazone, Penicillin, Cefoperazone/Sulbactam, and Spiramycin.^55^ The observed synergy between CaONPs and antibiotics is supported by the literature on bactericidal mechanisms of metal oxide nanoparticles. For instance, studies on ZnO nanoparticles show that combined treatments with antibiotics significantly improve membrane permeability and bacterial uptake.^56^ Although specific examples involving CaO are not available in the previous reports, the results of this study demonstrate that the physiological rationale is consistent across metal oxide–antibiotic combinations. The slight variations in antibacterial efficacy can be attributed to the differences in synthesis methods, particle size, and surface characteristics. *S. japonica*-mediated synthesis presented a viable and effective approach for producing CaONPs with notable antibacterial properties. The results also showed that calcination enhances the antibacterial efficacy of CaONPs. The nanoparticles alone exhibited good antibacterial activity but were less effective than the standard antibiotics. The combination of CaONPs with antibiotics significantly boosts their antibacterial efficiency, likely due to enhanced nanoparticle penetration and the disruption of bacterial membranes. These findings suggest that CaONPs, especially when used synergistically with antibiotics, could be promising antimicrobial agents for biomedical applications.

**Figure 9:**
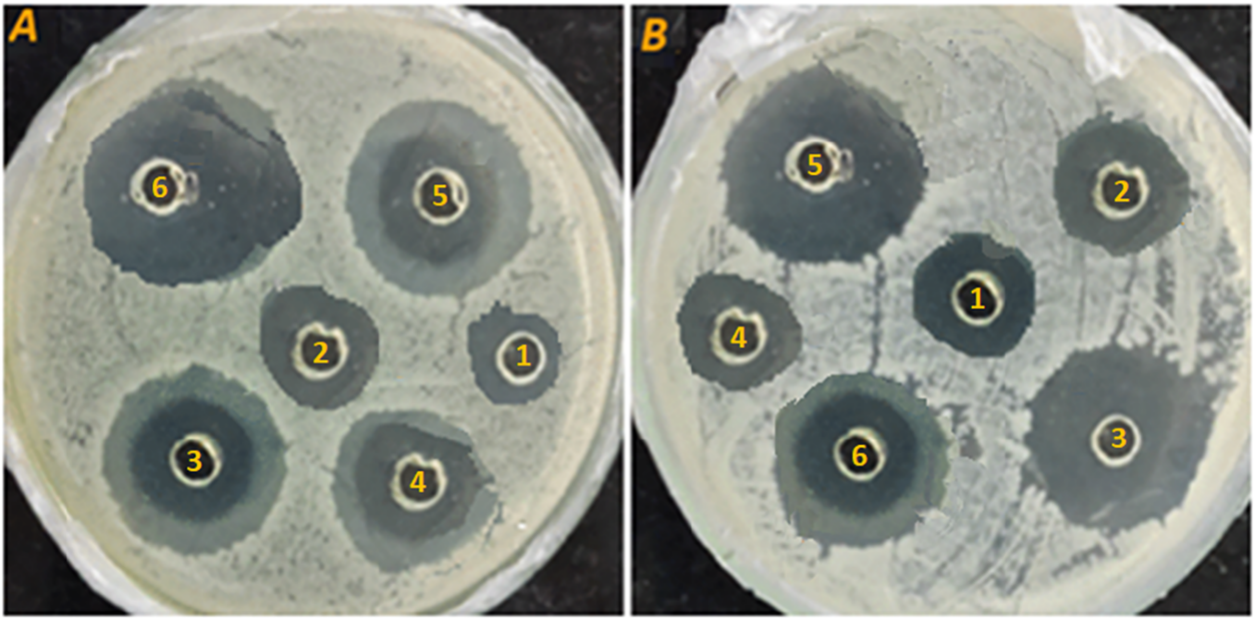
Antibacterial activity of *S. japonica*-mediated CaONPs against *E. coli* (A) and *S. aureus* (B). 1: CaONP I (Before calcination), 2: CaONP II (After calcination), 3: Streptomycin, 4: Ampicillin, 5: Streptomycin + CaONP II, and 6: Ampicillin + CaONP II.

**Table 2:** Antibacterial Activity of CaONPs against *E. coli* and *S. aureus*

zone inhibition (mm)			
		*E. coli*	*S. aureus*
nanoparticles	CaONP I	12 ± 1	15 ± 1
	CaONP II	16 ± 1	18 ± 2
antibiotics	Streptomycin	19 ± 1	21 ± 2
	Ampicillin	18 ± 1	14 ± 1
antibiotics + nanoparticles	Streptomycin + CaONP II	22 ± 2	24 ± 2
	Ampicillin + CaONP II	24 ± 2	19 ± 1

The antifungal activity of CaONPs was assessed against *C. albicans* and *A. niger*, both of which are known to cause opportunistic infections in humans. The results, presented in Table 3, show a significant improvement in the antifungal efficacy after calcination and in combination with standard antifungal agents. The inhibition zones were 14 ± 1 mm for *C. albicans* and 17 ± 1 mm for *A. niger* before calcination. After calcination, the inhibition zones increased to 18 ± 2 mm for *C. albicans* and 20 ± 2 mm for *A. niger*. The improved inhibition zones after calcination suggest that thermal treatment enhances the bioactivity of CaONP II, possibly due to increased crystallinity, surface area, and reactivity. The enhanced activity could also be attributed to the removal of organic residues from the precursor material, leading to purer and more reactive CaONPs. These results exceed those obtained from CaONPs synthesized by using *A. squamosa* seeds.^57^ Table 4 shows the types and antimicrobial properties of CaONPs reported by previous studies. When compared with standard antifungal drugs, Amphotericin B showed inhibition zones of 16 ± 1 mm for *C. albicans* and 17 ± 1 mm for *A. niger*. Clotrimazole exhibited slightly higher inhibition zones of 21 ± 1 mm for *C. albicans* and 18 ± 2 mm for *A. niger*. While CaONP II alone exhibited antifungal properties, its inhibition zones were slightly lower than those of clotrimazole but comparable to those of amphotericin B. This indicates that CaONP II possesses intrinsic antifungal activity, making them promising for biomedical applications. The combination of Amphotericin B + CaONP II resulted in 21 ± 2 mm for *C. albicans* and 23 ± 2 mm for *A. niger*. The combination of Clotrimazole and CaONP II showed even greater inhibition, reaching 27 ± 3 mm for *C. albicans* and 22 ± 2 mm for *A. niger*. These results indicate a synergistic effect where the antifungal efficacy of standard drugs was enhanced when combined with CaONPs. The increased inhibition zones suggest that CaONPs may enhance membrane disruption, increase oxidative stress, and facilitate better drug penetration into fungal cells. The antifungal activity of CaONPs is likely attributed to the generation of reactive oxygen species (ROS), which causes oxidative stress and damage to fungal cell walls and disruption of fungal membrane integrity, leading to leakage of essential intracellular components, an alkaline microenvironment that inhibits fungal growth by altering pH balance, or synergistic interactions with antifungal drugs, possibly improving drug delivery and efficacy. The results demonstrate that CaONPs exhibit significant antifungal activity against *C. albicans* and *A. niger*, with enhanced effects after calcination. The combination of CaONP II with Amphotericin B and Clotrimazole further improved their effectiveness, highlighting the potential of CaONPs as antimicrobial agents in combination therapy. The synergistic effects of CaONP II combined with Amphotericin B and Clotrimazole—producing inhibition zones up to 27 ± 3 mm—align with the literature on inorganic nanoparticle–antifungal synergy. In particular, inorganic nanoparticles have been shown to enhance Amphotericin B’s efficacy by inducing oxidative stress and disrupting fungal redox balance.^58^ Similar synergistic outcomes were reported with AuNPs combined with Amphotericin B, where nanoparticle-assisted biofilm disruption led to improved antifungal performance.^59^ Collectively, these comparisons emphasize that S. japonica-mediated CaONPs display intrinsic antifungal activity comparable to other green-synthesized metal oxides and, importantly, potentiate the action of conventional antifungal drugs—supporting their potential as dual-function agents in antimicrobial therapy.

**Table 3:** Antifungal Activity of CaONPs against *C. albicans* and *A. niger*

zone inhibition (mm)			
		*C. albicans*	*A. niger*
nanoparticles	CaONP I	14 ± 1	17 ± 1
	CaONP II	18 ± 2	20 ± 2
antifungals	Amphotericin B	16 ± 1	17 ± 1
	Clotrimazole	21 ± 1	18 ± 2
antifungals + nanoparticles	Amphotericin B + CaONP II	21 ± 2	23 ± 2
	Clotrimazole + CaONP II	27 ± 3	22 ± 2

**Table 4:** Types and Antimicrobial Properties of CaO Nanoparticles

nanoparticle	method	microbial stain	inhibition zone (mm) or key finding	references
CaO	Biosynthesis-waste eggshells	*E. coli*, *S. aureus*	19.46, 19.94	Banik, 2025 ^60^
CaO	Biosynthesis-plant	*E. coli*, *K. Pneumonia*, *B. subtilis*, *S. aureus*, *Methicillin-resistant* *S. aureus*, *Trichophyton rubrum*, *Aspergillus terreus*, and *Aspergillus flavus*	22, 13, 12,16, 12, 16, 14, 16	Ahmad, 2024 ^46^
CaO	Biosynthesis-plant	*E. coli* *, Bacillus sp*., *Pseudomonas sp*. *Aspergillus fumigatus*, *A. niger*, *Arthrographis cuboidea*	22.67 ± 0.5, 24.67 ± 0.5, 21.33 ± 0.5, 17.67 ± 0.5, 16.67 ± 0.5, 26.67 ± 0.5	Ahmad, 2024 ^61^
CaO	Biosynthesis-plant	*P. aeruginosa*, *S. aureus*, *K. pneumoniae*, *P. vulgaris*, *E. coli*	28 ± 1.0, 23 ± 0.3, 18 ± 0.9, 13 ± 1.6, 11 ± 0.5	Khan, 2023 ^62^
CaO	synthesis-chemical	Gr-positive and Gr-negative	effective in generating ROS, leading to bacterial cell death; *in silico* studies indicate potential efficacy against *E. coli*	Abbas, 2022,^63^ Harish, 2022 ^64^
CaO	synthesis-chemical	*E. coli*, *V. cholera*	exhibits antibacterial activity against *E. coli* and *V. cholerae*, suggesting potential for biomedical applications.	Kumar, 2021 ^33^
CaO	Biosynthesis-plant	*E. coli*, *S. aureus*	11.78, 12.65	Sultan, 2024 ^65^
CaO	Biosynthesis-plant	*E. coli*, *S. aureus*, *C. albicans*, *A. niger*	12 ± 1, 15 ± 1, 14 ± 1, 17 ± 1 (before calcination) 16 ± 1, 18 ± 2, 18 ± 2, 20 ± 2 (after calcination)	present study

### 3.5 *In Vitro* Drug Release Performance of CaONPs

In addition to antimicrobial applications, the potential of CaONPs as drug carriers was explored by analyzing the release profile of a model therapeutic agent, Zoledronic acid (ZA), under physiological and acidic conditions. The *in vitro* drug release profile of CaONPs adsorbed with ZA was studied at two different pH levels, 5.0 and 7.4, over a period of 250 h. The drug loading capacity of CaONPs was determined as 67.38%. The drug release performance of CaONPs exhibited a rapid release of ZA under both pH conditions at the beginning of the experiment (0–50 h), which is a common phenomenon due to the desorption of loosely bound drug molecules from the nanoparticle surface (Figure 10). The release at pH 5.0 was faster and higher compared to that at pH 7.4 in this phase, indicating that the acidic environment facilitates drug dissolution and diffusion. Beyond 50 h, the drug release continued but at a slower rate, showing a more controlled and sustained release pattern. The release profile at pH 5.0 remained consistently higher than that at pH 7.4 throughout the duration of the experiment. The cumulative release at pH 5.0 reached 92% at 250 h, whereas at pH 7.4, it stabilized at around 77%. This suggests that the CaONPs exhibited a pH-responsive drug release behavior with enhanced release in acidic conditions. There are three possible mechanisms behind the observed release behavior.^66^ The first one is the pH-dependent solubility. The higher release at acidic pH could be attributed to the increased solubility of CaONPs and the protonation of ZA, facilitating its faster diffusion. The second mechanism is nanoparticle degradation since CaONPs are known to degrade more rapidly in acidic environments, leading to a faster release of the adsorbed drug. The third possible mechanism is related to electrostatic interactions. The charge of the nanoparticle surface and the drug molecules may vary with pH, and this influences the binding strength and desorption rate. The enhanced release at pH 5.0 suggested that these CaONPs could be effective for targeted drug delivery in acidic environments, such as cancerous tissues or bone resorption sites. The controlled release at pH 7.4 indicated stability under physiological conditions, making them suitable for systemic drug administration. pH-responsive drug delivery systems are designed to detect and respond to pH changes within the body, allowing for precise drug release at target sites.^67^ These systems often utilize materials that undergo structural or solubility changes in response to specific pH levels. In the context of CaONPs, acidic environments can induce protonation of functional groups or hydrolysis of acid-labile bonds within the nanoparticle matrix and lead to accelerated drug release. Whereas the nanoparticles may remain more stable, resulting in a slower and sustained drug release in neutral environments, such as physiological pH (pH 7.4). This pH-responsive behavior ensures that the drug is released more rapidly in the acidic microenvironments of tumors or inflamed tissues, while maintaining stability in normal physiological conditions.^68^ Utilizing pH-responsive mechanisms in drug delivery offers several benefits such as enhanced targeting specificity, reduced systemic toxicity, and controlled release rates. Drugs are preferentially released in diseased tissues with characteristic pH levels, thereby reducing off-target effects. Exposure to healthy tissues is minimized by limiting drug activation to target sites, thus decreasing potential side effects. The drug release rate can be fine-tuned based on the pH sensitivity of the carrier, which allows for sustained therapeutic effects. These advantages contribute to more effective and safer therapeutic outcomes. Nanoscale drug delivery systems utilizing ZA have demonstrated similar pH-responsive release behaviors. For example, a PEGylated chitosan/PLGA nanoparticle formulation exhibited minimal ZA release (<5%) at physiological pH over 48 h, but significantly increased release under acidic tumor-like conditions.^69^ Likewise, a tumor acidity-responsive polymeric nanoparticle system designed for ZA delivery revealed that PEG detachment and surface charge conversion in acidic environments substantially increased intracellular uptake and antitumor efficacy compared to neutral conditions.^70^ These findings corroborate the results of this study and underscore that *S. japonica*-mediated CaONPs enable acid-triggered drug delivery—offering targeted release profiles (92% at pH 5.0 vs 77% at pH 7.4) suitable for tumor microenvironments. The pH-dependent release profile of ZA from CaONPs demonstrates the potential of these nanoparticles as effective carriers for targeted drug delivery. Such systems can enhance drug efficacy while minimizing adverse effects by leveraging the pH variations between healthy and diseased tissues, aligning with the goals of advanced nanomedicine strategies.

**Figure 10:**
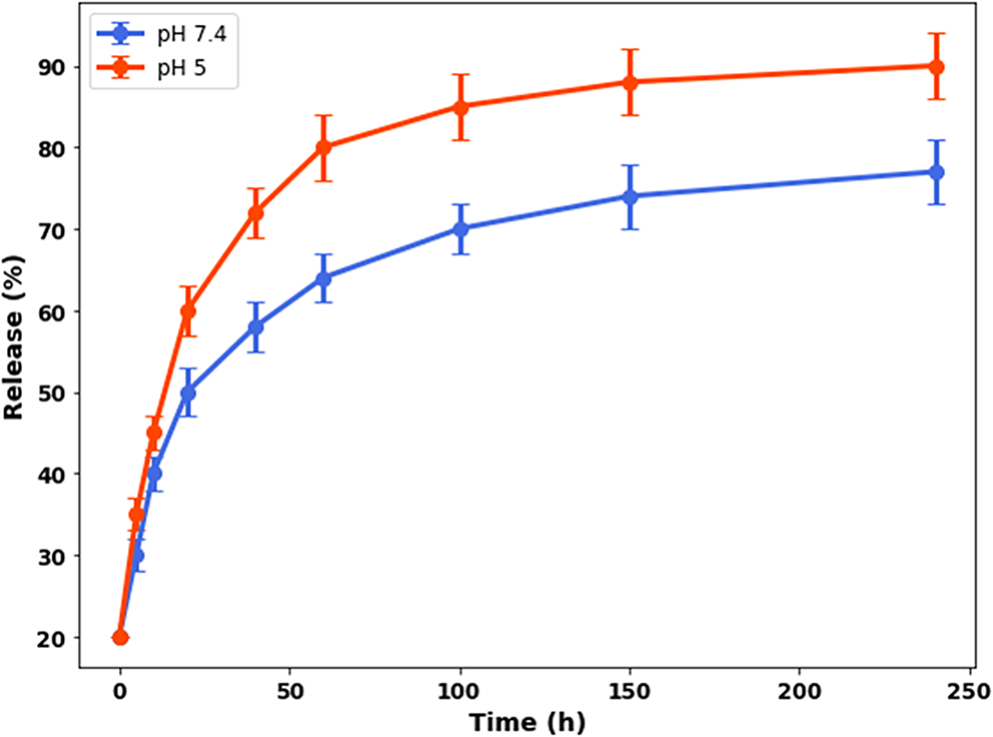
*In vitro* drug release performance of CaONPs adsorbed with Zoledronic acid at pH 5.0 and 7.4.

### 3.6 Cytotoxicity of CaONPs

To assess the biocompatibility of the synthesized CaONPs for drug delivery and therapeutic use, cytotoxicity was evaluated using Saos-2 osteosarcoma cells. The MTT assay results indicated that the CaONPs synthesized via the green method did not exhibit significant cytotoxic effects on the Saos-2 cell line within the tested concentration range. Based on the values in the Figure 11, CaONPs showed dose-dependent effects in the Saos-2 cell line and were not cytotoxic, especially at low concentrations (96–116% cell viability). Cell viability was observed to decrease at high concentrations (78.25–82%), but these rates did not reach the full toxicity level. At a concentration of 125 μg/mL, the viable cell percentage was 92% ± 2.83, which possibly represents a safer limit range. It also reached higher levels than the control group, meaning that low doses support cell metabolism or promote cell proliferation. This rate decreased to 79% ± 2.65 in the concentration of 250 μg/mL. Cell viability was 78.25% ± 0.96 at the highest concentration (1000 μg/mL), which still suggested that the nanoparticles were not overtly toxic. As the concentration decreased, cell viability gradually increased, reaching 96.00% ± 1.41 at 62.50 μg/mL and exceeding control levels at the lowest concentration (31.25 μg/mL, 116.00% ± 2.83), indicating potential stimulatory or proliferative effects. These findings suggested that CaONPs are biocompatible and do not elicit cytotoxic responses in Saos-2 cells. Moreover, the observed increase in cell viability at lower concentrations may reflect enhanced cellular metabolic activity or proliferation, which has also been reported for other metal oxide nanoparticles in the literature. For example, studies on zinc oxide (ZnO) and titanium dioxide (TiO_2_) nanoparticles have demonstrated similar dose-dependent responses, with low concentrations promoting cell growth while higher concentrations show mild cytotoxicity. A study on TiO_2_ nanoparticles reported reduced fibroblast cell viability and induced reactive oxygen species (ROS) production at higher concentrations (150 and 250 μg/mL), while lower concentrations did not affect cell viability.^71^ Similarly, ZnO nanoparticles have been shown to induce cytotoxicity in a dose-dependent manner, with higher concentrations leading to increased DNA damage and oxidative stress.^72^ Overall, the concentrations tested in this study may be optimal doses in biomedical applications compatible with bone cells. These doses can be considered biocompatible since they both show cell viability enhancing effects and do not cause cytotoxicity. These results confirm the safety and potential applicability of the CaONPs in biomedical contexts, particularly when used at lower concentrations. Similar dose-dependent cytotoxicity profiles have been reported for other metal oxide nanoparticles. Maringgal et al. (2020) reported a significant (*P* < 0.05) cytotoxic effect of CaONPs mediated by *Trigona* sp. honey at 500 g/mL concentration, whereby the viability of 65.55 and 59.77% was observed for MRC 5 cells and VERO cells after 72 h of exposure, respectively.^73^ Eram et al. (2021) investigated CaONPs synthesized using *Crescentia cujete* leaf extract and demonstrated excellent cell survival in zebrafish models at concentrations ≤80 μg/mL, with an LC_50_ of 86.3 μg/mL, suggesting low cytotoxicity at sublethal exposures.^39^ More broadly, the nanoparticle biocompatibility literature consistently shows a hormetic effect: low doses may stimulate cell metabolism or proliferation, while higher doses induce mild cytotoxicity.

**Figure 11:**
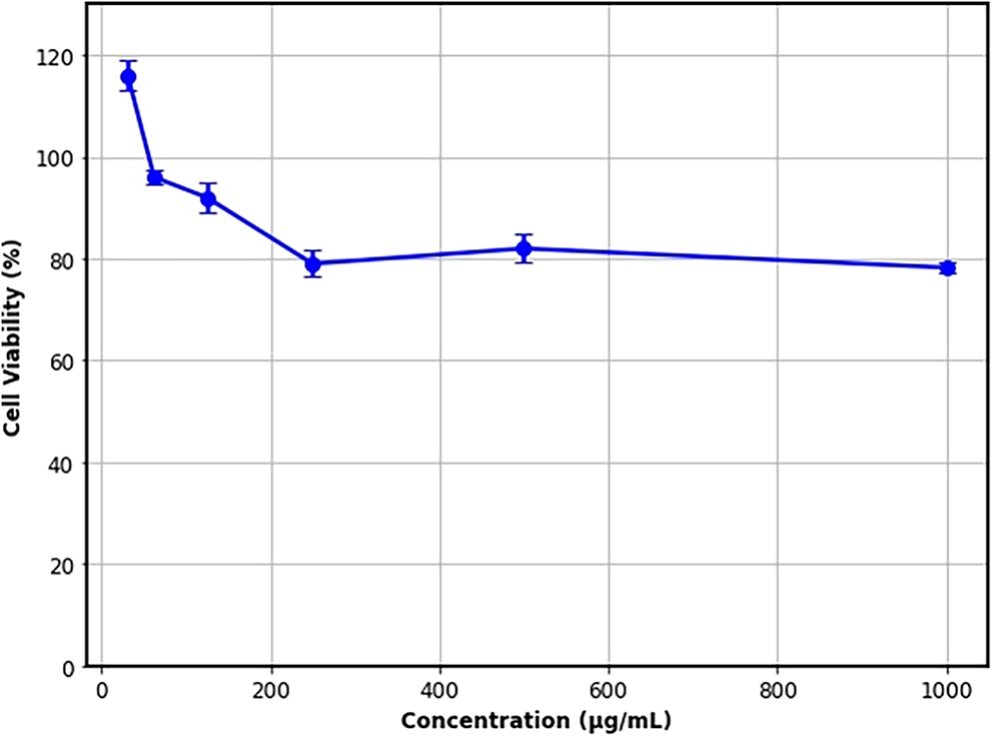
*In vitro* cytotoxicity of synthesized CaONPs on the Saos-2 cell line after 24 h exposure.

The noncytotoxic nature of CaONPs synthesized in this study, combined with high biocompatibility, makes them highly promising for various medical applications related to bone cells. They can be used as scaffold materials in bone tissue engineering that support cell attachment, proliferation, and differentiation by facilitating bone regeneration. Their ability to release calcium ions in a controlled manner can stimulate osteoblast activity and accelerate bone matrix formation, making them suitable for bone grafts and fracture healing. Additionally, their reported antibacterial properties can be leveraged in implants or coatings to reduce infection risks while maintaining compatibility with bone cells. CaONPs also hold potential in drug delivery systems, enabling the targeted release of therapeutics for bone diseases and minimizing systemic side effects. Furthermore, they can be utilized in biosensors to monitor bone metabolism or diagnose diseases. They can enhance the surface integration with bone tissue while preventing infections in orthopedic implants. Lastly, their nontoxic profile makes them an ideal candidate for drug delivery systems targeting bone tumors, as they can deliver therapeutics without harming normal bone cells.

### 3.7 Dye Degradation Performance of CaONPs

Beyond biomedical applications, the environmental utility of CaONPs was investigated through dye degradation studies using Congo red as a model pollutant to assess their potential in wastewater treatment. The Congo red degradation efficiency using CaONPs at two different pH levels (5.0 and 9.0) at 25 °C between 2 min and 24 h is illustrated in Figure 12A. It can be seen from the graph that the dye removal percentage was significantly greater in an alkaline environment (pH 9.0) compared with the acidic condition (pH 5.0). At pH 9.0, the degradation was 41% at the early stage (2 min) and showed a rapid increase after 30–45 min. The degradation reached 84% in 24 h. At pH 5.0, the degradation was much slower, starting at 18% and only reaching about 34% at 3 h and finally reaching 78% after 24 h. CaONPs showed a time-dependent degradation behavior, where in both cases, dye degradation increased progressively over time. At pH 9.0, a faster degradation rate was observed in the first 45 min to 1 h, which then continued to increase more gradually. At pH 5.0, the degradation occurred at a much slower rate, with only a slight increase until the 3 h mark, followed by a more noticeable increase at 24 h. pH 9.0 facilitated stronger dye degradation, likely due to enhanced hydroxyl ion (OH^–^) concentration, promoting oxidative degradation through hydroxyl radicals (^•^OH) or electrostatic interactions between CaONPs and Congo red were more favorable in an alkaline medium, improving adsorption and subsequent degradation. Lower degradation at pH 5.0 may be due to protonation of active sites on CaONPs, reducing their ability to generate hydroxyl radicals or less effective dye-nanoparticle interactions, leading to reduced degradation efficiency. At acidic pH, the structure of Congo red is protonated (especially −NH_2_ group → −NH_3_^+^), which makes the net charge of the molecule less negative, may reduce π–π interactions and surface binding, and may decrease adsorption. At basic pH, the structure of the molecule remains more stable and ionically active (fully anionic), interacting better with groups on the CaO surface. The dye degradation analysis highlights the importance of optimizing the pH for maximum degradation efficiency when using CaONPs in wastewater treatment applications.

**Figure 12:**
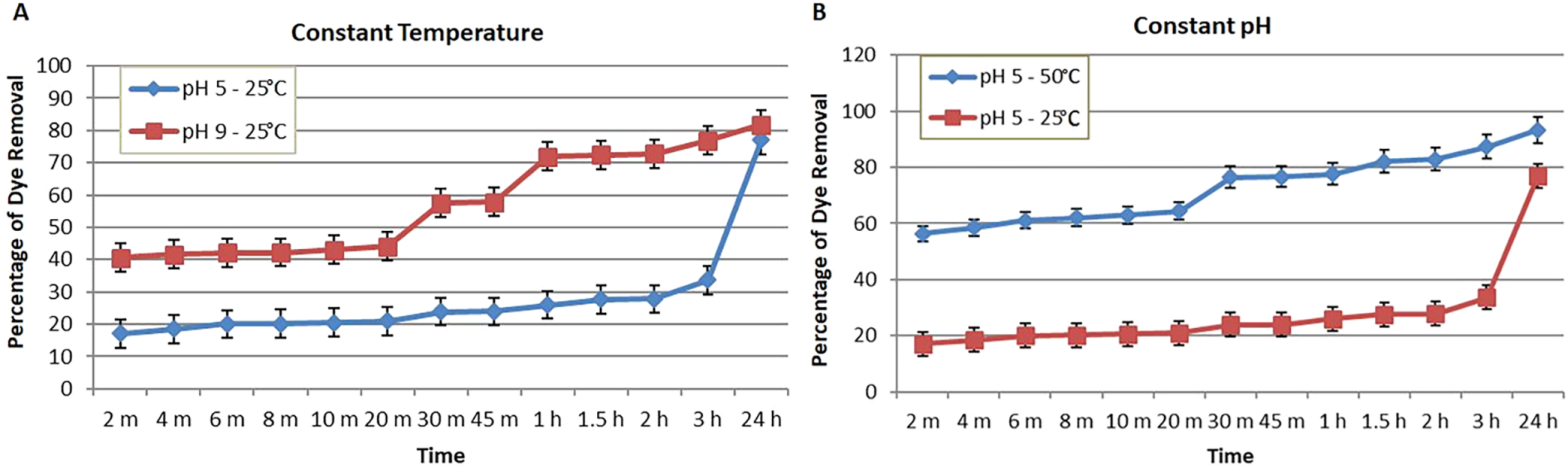
Dye removal performance of CaONPs using Congo red at a constant temperature (A). Dye removal performance of CaONPs using Congo red at constant pH (B).

Figure 12B illustrates the percentage of Congo red removal over time using CaONPs at pH 5.0 under two different temperatures, 50 and 25 °C. At 50 °C, the dye removal percentage of CaONPs was 58% in 2 min, and it gradually increased, reaching 93% after 24 h. In contrast, the removal efficiency remained significantly lower at 25 °C, starting at 18% and only reaching around 79% after 24 h. The graph shows that higher temperature enhances dye removal efficiency. The removal rate was consistently higher at 50 °C, showing a steady increase with a sharper rise beyond 30 min. At 25 °C, the removal efficiency remained nearly constant for the first 3 h, showing only a minor increase, but then a significant jump at 24 h. Temperature also influenced the adsorption and degradation of Congo red to CaONPs. The higher removal efficiency at 50 °C suggested that elevated temperature enhances adsorption and/or degradation kinetics. Higher temperatures generally improved molecular diffusion and surface interactions, leading to faster dye degradation. The lower performance at 25 °C implied slower reaction kinetics, requiring longer contact time for substantial degradation. CaONPs exhibited significantly higher efficiency at elevated temperatures. This behavior aligns with the temperature-dependent nature of adsorption, where increased thermal energy accelerates the reaction. Low pH conditions such as pH 5.0 might influence the surface charge of the nanoparticles and enhance electrostatic interactions with Congo red molecules. Overall, CaONPs showed superior Congo red dye removal at 50 °C compared to 25 °C. The removal efficiency was much higher and faster at 50 °C, demonstrating that temperature plays a crucial role in enhancing the adsorption and degradation kinetics of dye molecules. The dye removal performance of CaONPs synthesized via green methods has been a subject of recent research due to the environmental benefits and efficiency of these methods. For instance, a study utilized CaONPs mediated from *Morus nigra* leaf extract in removing Rhodamine B from aqueous solutions and the nanoparticles achieved a 98% removal efficiency at 70 °C, demonstrating results parallel to those reported in this study.^74^ Menezes et al. (2024) reported 76.2% photodegradation of yellow tartrazine at pH: 7.0 using CaONPs synthesized from *Linum usitatissimum* L. extract; however, their system required visible light activation, whereas findings in this study show similarly high removal rates via purely adsorptive processes.^75^ Thakur *et al*. (2021) synthesized CaO nanospheres from eggshell waste and reported 98% adsorption of Brilliant green and 78% removal of Phenol red in binary systems, with strong electrostatic interactions playing a key role.^76^ While Thakur *et al*. focused on binary mixtures of dye types, this study extends these findings by demonstrating comparable or superior removal efficiencies for a single dye, Congo red, under simple adsorption conditions. Some of the previous studies reported in the literature are listed in Table 5. The similarity in removal efficiency across different dye chemistries and synthesis methods highlights the versatility and robustness of green-synthesized CaO nanoparticles for water treatment.

**Table 5:** Types and Dye Degradation Performances of the CaO Nanoparticles

nanoparticle	method	dye degradation (%)	comment	references
CaO	Biosynthesis-plant	95.17	adsorption of Rhodamine B. dye concentration of 80 ppm, 0.4 g nanoparticles (after 150 min).	Nasir, 2024 ^74^
CaO	Biosynthesis-plant	76.2	photodegradation of CCRD 23, the ideal condition was pH = 7.0, [CaONPs] = 1.2 g/L, and [YT] = 20 mg/Lqq	Menezes, 2024 ^75^
CaO	green synthesis-*Crassostrea gigas* shells	75	Bromocresol green dye (BCGD) photodegradation under sun radiation	Ogoko, 2024 ^77^
CaO	Biosynthesis-plant	64, 53, 49	photocatalytic degradation of methyl red, methyl orange, and methylene blue. concentration of NPs: 125 μg/mL	Brindhadevi, 2024 ^78^
CaO	Biosynthesis-plant	reduction in the absorption between 350 and 500 nm.	to 100 mL of 25 ppm concentrated Congo red, 100 mg of the CaONPs	Anantharaman, 2016 ^79^
CaO	green synthesis-waste eggshells	72	microwave-assisted catalytic degradation of malachite green at a concentration of 50 ppm in the presence of 100 mg of CaO nanoparticles within 30 min	Zia, 2025 ^80^
CaO	Biosynthesis-plant	90	the photodegradation of methylene blue (MB) dye, MB dye (10^–6^ M), and 30.0 mg of the CaONPs (after 180 min)	Moghaddas, 2024 ^81^
CaO	Biosynthesis-plant	58 in 2 min, 93 in 24 h	adsorption of Congo red, 0.1 mg/mL concentration of dye mixed with 20 mg of CaONPs	present study

## 4 Conclusions

This study demonstrated the successful green synthesis of calcium oxide nanoparticles (CaONPs) from *S. japonica* and their multifunctional potential in environmental and biomedical domains. The results of the *in silico* docking study suggested that CaONPs possess strong and stable binding affinities toward multiple bacterial outer membrane proteins, in some cases exceeding those of clinically approved antibiotics. Additionally, CaO showed comparable binding performance to Zoledronic acid against the FDPS enzyme, indicating its potential dual functionality as both an antimicrobial and a bone-targeted agent. These findings supported further exploration of CaONPs as multifunctional nanomaterials in biomedical applications, particularly for addressing microbial resistance and bone-related disorders. Moreover, molecular docking results highlighted specific interactions between CaONPs and key amino acid residues located within functional domains of bacterial outer membrane proteins. Integrating these findings with prior literature further reinforces the promising biomedical relevance of CaONPs. Key findings of this study also include synergistic effects of CaONPs combined with antibiotics, particularly Ampicillin and Streptomycin, indicating their potential to combat resistant strains. *In vitro* studies demonstrated a pH-sensitive sustained release of Zoledronic acid (92% release at pH 5.0). The nanoparticles maintained high cell viability (≥78%) in Saos-2 cells at concentrations between 31 and 1000 μg/mL, highlighting their noncytotoxic nature. Effective Congo red degradation was obtained under both alkaline and acidic conditions (up to 93% removal). The novel contribution of this study is the first-time use of *S. japonica* extract for CaONP synthesis, combined with an integrated evaluation of their antimicrobial, adsorptive, and drug delivery properties supported by both *in vitro* and *in silico* analyses. These findings highlight the promising potential of CaO nanoparticles as effective and safe candidates for targeted cancer therapy, smart drug delivery systems, medical interventions, such as infection prevention, and even environmental remediation efforts. However, further studies are needed to confirm their practical applicability of the findings, such as *in vivo* validation, broader cytotoxicity testing, and assessment of long-term stability. Overall, the multifunctionality of CaONPs, spanning environmental remediation, drug delivery, antimicrobial activity, and biocompatibility, underscores their potential in various industrial and biomedical applications.
